# Relationship Between Live Body Weight and Linear Body Measurements in Goats: A Meta-Analysis Using a Multilevel Random-Effects Model

**DOI:** 10.3390/ani16193107

**Published:** 2026-10-02

**Authors:** Thobela Louis Tyasi, Gaetan Kabera

**Affiliations:** 1 Department of Statistics, University of South Africa, Pretoria 0002, South Africa; kaberg@unisa.ac.za; 2 Department of Agricultural Economics and Animal Production, University of Limpopo, Sovenga 0727, South Africa

**Keywords:** goats, body weight, heart girth, withers height, body length

## Abstract

This study determined the relationship between live body weight (BW) and linear body measurements, including heart girth (HG), body length (BL) and withers height (WH) using a meta-analytic method. This meta-analysis synthesized the evidence from studies conducted in goats to examine the relationship between BW and HG, BL, and WH. Five electronic databases (Web of Science, Google Scholar, PubMed, OpenAlex, and Scopus) were systematically searched for studies published between 2005 and 2025. The Joanna Briggs Institute (JBI) tool was used to assess the risk of bias, and a multilevel random-effects model was used for analysis due to many studies containing multiple effect sizes. A total of 45 studies were included, with the sample size ranging from 30 to 1072 goats. The multilevel random-effects model revealed a pooled correlation coefficients of 0.83 between BW and HG (95% CI: 0.79–0.87), 0.79 between BW and BL (95% CI: 0.74–0.83), and 0.72 between BW and WH (95% CI: 0.64–0.78). The results also revealed a pooled regression coefficients of 0.65 (95% CI: 0.55–0.74), 0.40 (95% CI: 0.04–0.76), and 0.06 (95% CI: −0.02–0.14) for HG, BL and WH, respectively. This meta-analysis provides evidence of strong positive significant associations between body weight and heart girth, body length, and withers height in goats. The findings highlight the potential of these linear body measurements as practical indicators of body weight, particularly in production systems where weighing equipment is not available.

## Simple Summary

This study aimed to examine the relationship between live body weight and linear body measurement traits, including heart girth, body length and withers height, utilizing a multilevel random-effects model. The study used Web of Science, Google Scholar, PubMed, OpenAlex, and Scopus to search for articles and included forty-five studies published between 2005 and 2025. The findings revealed evidence that heart girth, body length and withers height had a strong positive relationship with body weight in goats.

## 1. Introduction

In animal breeding, the use of linear body measurements has been reported as a suitable approach to estimate the live body weight of animals since they are mostly positively correlated with live body weight [1,2,3]. However, goat farmers find it difficult to understand which linear body measurements might be used as practical indicators of live body weight in the absence of a weighing scale during management practices such as marketing, vaccinations, and selection for breeding purposes [4]. The problem is that there is controversy in findings from different studies that makes it difficult for researchers, agricultural advisers, and agricultural extension officers to advise goat farmers on the use of linear body measurements for estimating live body weight. Goat farmers may use linear body measurements to estimate body weight as the cheapest practice compared to buying weighing scales [3]. However, it is important to determine the linear body measurements that would appropriately replace a weighing scale. Goat farmers lack a weighing scale for measuring their goats’ weight [2]. Meta-analysis is a good statistical method for contradictory findings to produce one decision [5]. The objective of the study was to determine the relationship between body weight and heart girth, body length, and withers height using the meta-analysis approach. Addressing this gap is crucial for the accuracy of live body weight estimation in animal management procedures.

## 2. Materials and Methods

### 2.1. Study Design

**Figure 1:**
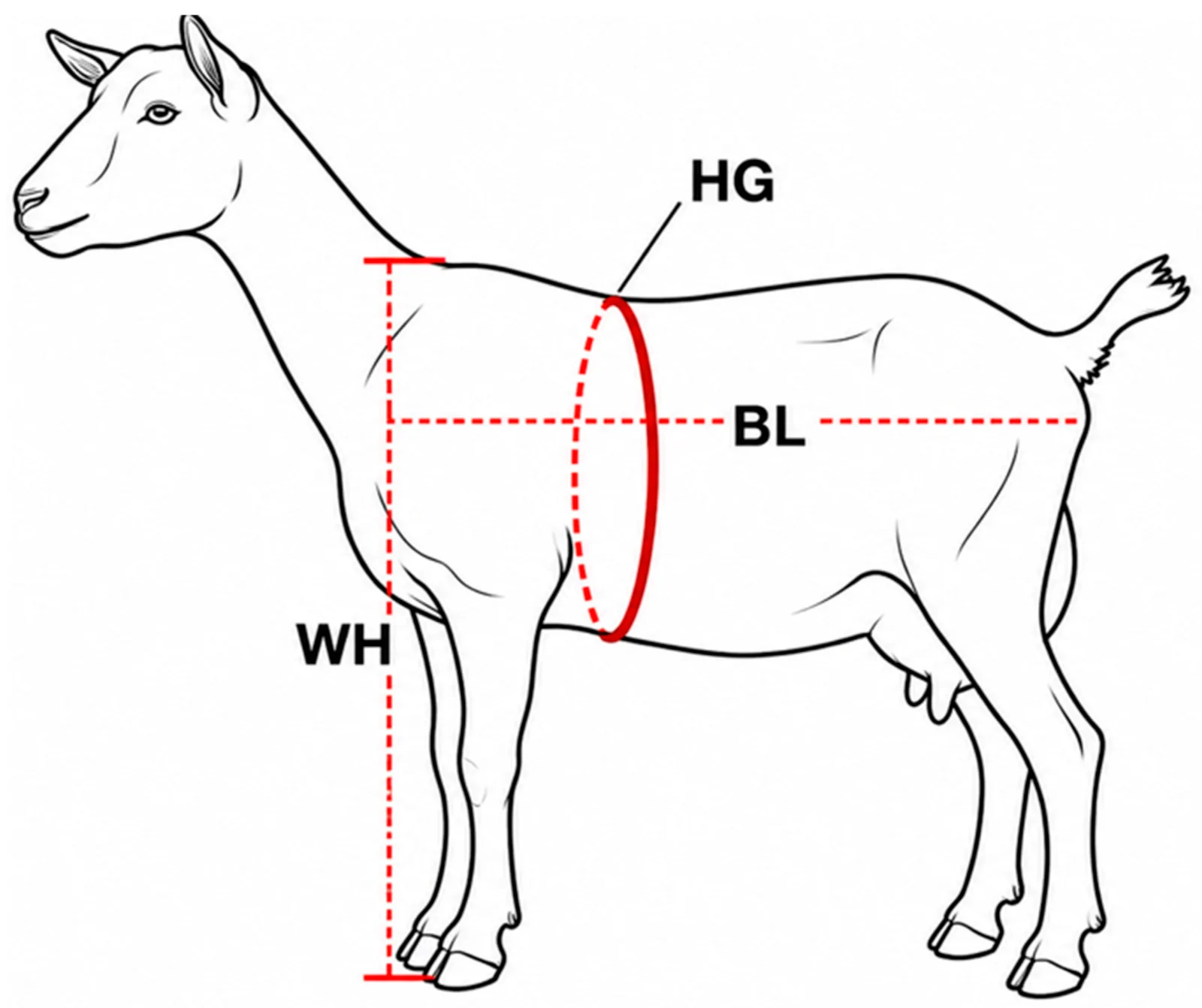
Diagram illustrating the linear body measurements. HG: heart girth, BL: body length, WH: withers height.

A meta-analysis was conducted to quantitatively synthesize published evidence on the relationship between live body weight and linear body measurements in goats. The study focused on three commonly used body measurements: (1) heart girth (HG), which is measured as the perimeter of the chest just behind the front legs and withers; (2) body length (BL), which is measured as a distance from the point of the shoulder to the pin bone; and (3) withers height (WH), which is measured as the vertical distance from the ground at the fore hoof to the point of the ridge between the shoulder bones. Figure 1 illustrates the measurement procedure for HG, BL and WH.

### 2.2. Eligibility Criteria

The Population, Exposure, Outcome, and Study Design (PEOS) framework was used for eligibility. Population (P): We included studies on domestic goats (Capra hircus) of any breed, age, sex, or production system. Studies on other species were excluded. Exposure (E): We included studies with at least one linear body measurement, including but not limited to heart girth (HG), body length (BL), and withers height (WH). All linear body measurements were measured in centimetres. Outcome (O): Studies including direct live body weight (BW) or body weight estimation/prediction models were also included. The BW was measured in kilograms in all the articles. Studies focusing solely on carcass traits, body condition scoring without estimating body weight and its relationship with linear body measurements were excluded. Study design and data (S): Primary empirical research articles reporting quantitative relationships using Pearson’s correlation coefficients and linear regression models, or prediction models for estimation of body weight from linear body measurements such as HG, BL and WH, were included. Publication type and language: Peer-reviewed full-text journal articles published in English were included. Conference abstracts, review papers, and articles with incomplete or non-extractable statistical data were excluded.

### 2.3. Search Strategy

The systematic search was conducted across PubMed, Google Scholar, Web of Science, OpenAlex, and Scopus to identify studies assessing the prediction of live body weight in goats using linear body measurements. Searches were conducted up to 29 August 2025 without restriction on date. The search strategy was constructed around four primary concept groups joined by the AND operator: (1) target species (“goat”, “goats”, “caprine”); (2) outcome variable (“body weight”, “live weight”, and “body weight prediction”); (3) body measurement predictors (“heart girth”, “chest girth”, “thoracic girth”, “linear body measurements”, “body length”, and “withers height”); and (4) analytical methods (“correlation”, “regression”, and “prediction”). The individual terms were combined using the OR operator within each group. The full database-specific search strings, syntax rules, search limits, and total records retrieved for each database are detailed in Supplementary Table S1.

### 2.4. Inclusion Criteria

Titles and abstracts retrieved from the searched articles were screened manually to identify potentially relevant studies. Full-text screening was subsequently conducted, and studies were included if they: studied goats; reported body weight; reported at least one of the linear body measurements such as heart girth, body length and withers height; reported Pearson’s correlation coefficients between body weight and linear body measurements; reported regression coefficients predicting body weight from one of the linear body measurements such as heart girth, body length and withers height; reported sample size; were published in peer-reviewed journals; and were written in English.

### 2.5. Exclusion Criteria

Some of the studies were excluded during screening. The studies were excluded if they: did not include goats; did not report body weight data; did not report heart girth, body length, or withers height; lacked sufficient statistical information for effect size calculation; lacked correlation or regression coefficients; or were published in languages other than English due to resource constraints regarding certified technical translation to avoid potential misinterpretation of terminology for statistical and linear body measurements.

### 2.6. Article Selection Process

All records retrieved from the databases were exported and imported into Microsoft Excel for management. To minimise selection bias, the study selection process was conducted systematically in three sequential stages independently by the two co-authors (TLT and GK) following the pre-established PEOS eligibility criteria: Stage 1: Title screening, where titles were examined for relevance to body weight estimation or the relationship between body weight and linear body measurements in goats. Stage 2: Abstract screening, where abstracts were reviewed against the eligibility criteria to make sure all the relevant articles were included. Stage 3: Full-text screening, where full-text articles were evaluated for final eligibility.

**Figure 2:**
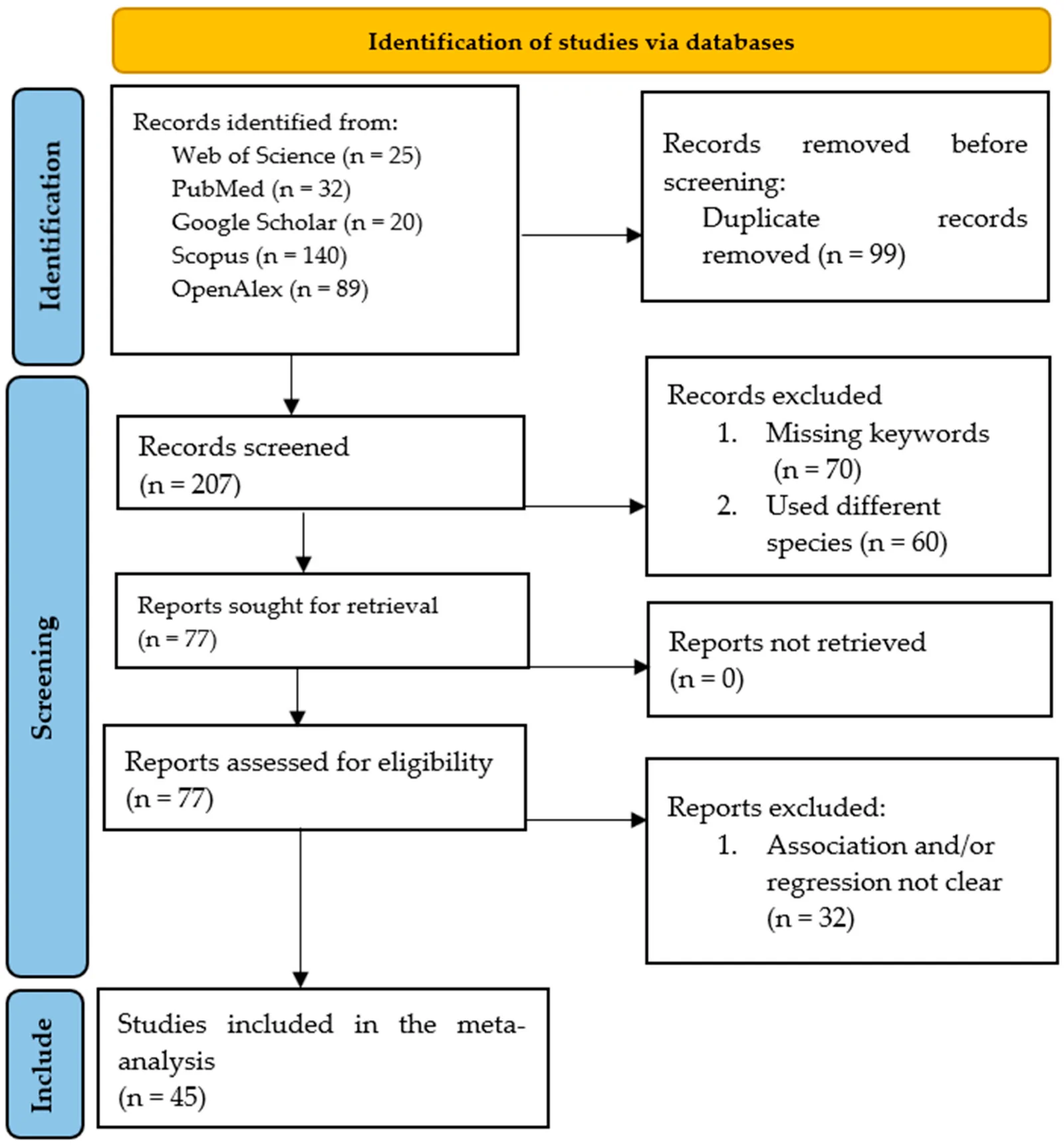
Flow chart of the identification and selection of studies used in the systematic review and meta-analysis.

Inter-reviewer disagreements regarding study inclusion at any screening stage were initially addressed through direct consensus discussions between the two co-authors. The complete selection workflow is presented in the PRISMA 2020 flow diagram (Figure 2).

### 2.7. Assessment of Risk of Bias

Joanna Briggs Institute (JBI) tool [6] was used to assess the quality of the articles selected in this study. The checklist consists of eight questions, namely: (1) Were the criteria for inclusion in the sample clearly defined? (2) Were the study subjects and the setting described in detail? (3) Was the exposure measured validly and reliably? (4) Were the standard criteria used for measurement of the condition? (5) Were confounding factors identified? (6) Were strategies to deal with confounding factors stated? (7) Were the outcomes measured validly and reliably? (8) Was appropriate statistical analysis used? Individual questions were examined for each selected article, and answers were given as ‘Yes’ or ‘No’. Overall risk was specified with two main answers: ‘Low’ and ‘Moderate’.

### 2.8. Data Extraction

The data for analysis were extracted manually from included studies. The following variables were recorded for each study: author(s) who wrote the article, year of publication, country where the study was conducted, goat breed, continent where the study was conducted, sex of the used goats, age of the used goats, sample size, heart girth, body length, withers height, Pearson correlation coefficient, regression coefficient type (standardised vs. unstandardised), standard error, regression model type, adjustment variables, variable units, regression intercept, coefficient of determination, confidence interval, and corresponding population.

### 2.9. Extraction of Effect Sizes

The correlation coefficients and regression coefficients were extracted from the included studies as the two types of effect sizes and analysed separately. Where a study reported more than one relevant effect size, all of them were retained for use.

### 2.10. Meta-Analysis of Correlation Coefficients

The effect sizes from included studies were analysed separately for each linear body measurement and transformed using the following Fisher’s *z* transformation:

$$z_{i}=\frac{1}{2}\ln\left(\frac{1+r_{i}}{1-r_{i}}\right)$$

where *r_i_* is the correlation coefficient reported by study *i*, and *z_i_* is the corresponding Fisher’s z-transformed effect size. The sampling variance of each transformed correlation coefficient was calculated as:

$$v_{i}=\frac{1}{n_{i}-3}$$

where *v_i_* is the sampling variance of the Fisher’s *z*-transformed correlation coefficient for effect size *i*, *n_i_* is the sample size of study *i*, and 3 is the adjustment associated with the variance of Fisher’s *z*.

The pooled Fisher’s z estimates were back-transformed to correlation coefficients for interpretation using the following equation:

$$r=\frac{e^{2z}-1}{e^{2z}+1}$$

where *r* is the back-transformed Pearson correlation coefficient, *z* is the pooled Fisher’s *z* estimate and *e* is the base of the natural logarithm.

### 2.11. Meta-Analysis of Regression Coefficients

The effect sizes were analysed separately for each linear body measurement. The effect sizes and standard errors (*SE_i_*) were used for analysis. The corresponding sampling variance was calculated using the following equation:

$$v_{i}=SE_{i}^{2}$$

where *v_i_* is the sampling variance of the *i*-th effect size, *SE_i_* is the standard error of the *i*-th effect size, and $SE_{i}^{2}$ is the squared standard error, giving the sampling variance.

### 2.12. Multilevel Random-Effects Model

The multilevel random-effects model was used because several studies contributed multiple effect sizes. The following model was used:

*y_ij_* = *μ* + *u_j_* + *e_ij_*

where *y_ij_* is the observed effect size *i* from study *j*, *μ* is the overall pooled effect, *u_j_* is the random effect associated with study *j*, and *e_ij_* is the sampling error of the effect size *i* within study *j*.

The model therefore accounts for the clustering of multiple effect sizes within a study using the following equation:

*y* = *Xβ* + *Zu* + *ε*

where *Xβ* is the fixed overall mean effect, *u* is the study-level random effects, and *ε* is the within-study sampling error.

### 2.13. Heterogeneity Analysis

Between-study heterogeneity was assessed using the estimated random-effects variance and Cochran’s Q statistic. The proportion of total variability attributable to heterogeneity was assessed using *I*^2^. *I*^2^ statistic was calculated using the following equation:

$$I^{2}=\frac{\tau^{2}}{\tau^{2}+\overset{`}{v}}\times 100(\%)$$

where *I*^2^ is the total variability in the observed effect sizes that is attributable to between-study heterogeneity rather than sampling error, *τ*^2^ is the estimated between-study variance, and $\overset{`}{\nu }$ is the average sampling variance of the effect sizes.

The significance of the moderators in the meta-regression models was assessed using the QM statistic, with the corresponding degrees of freedom and *p*-value reported.

### 2.14. Prediction Interval

A 95% prediction interval was calculated to estimate the range within which the true effect of a future comparable study would be expected to fall, taking between-study heterogeneity into account. This was reported alongside the pooled effect and 95% confidence interval because a confidence interval describes uncertainty around the pooled mean effect.

### 2.15. Forest and Funnel Plots

The forest plots were generated to display the individual effect sizes for the included studies per analysis, their 95% confidence intervals, and the pooled random effects estimate for linear body measurements. Funnel plots were used to visually assess the distribution of effect sizes against their precision and to identify potential asymmetry that could indicate small-study effects.

### 2.16. Small-Study Effects

Small-study effects were assessed using statistical tests of funnel-plot asymmetry. The multilevel approaches were considered to account for the dependence arising from multiple effect sizes contributed by the same study. Robust inference was used to account for clustering at the study level.

### 2.17. Meta-Regression

Univariable and multivariable meta-regression analyses were conducted to investigate whether study-level characteristics explained variation in the observed effect sizes in the correlation dataset. The meta-regression was not performed for the regression dataset due to the limited number of studies included per analysis. The following meta-regression model was used for the correlation dataset:

*y_i_* = *β*_0_ + *β*_1_*X*_1*i*_ + *β*_2_*X*_2*i*_ + ⋯ + *u_j_* + *e_i_*

where *X*_1_,*X*_2_, …, *X_p_* are the study-level moderators.

The statistical significance of the moderators was assessed using the QM test, with the corresponding degrees of freedom and *p*-values reported.

### 2.18. Sensitivity Analysis

The robustness of the pooled estimates was assessed using leave-one-publication-out sensitivity analysis. All effect sizes from one study were removed at a time, and the multilevel model was refitted. The sensitivity analysis was not performed for the regression dataset due to the limited number of studies used for analysis.

### 2.19. Statistical Analysis

All statistical analyses were conducted using the metafor package in R version 4.5.2 [7] (R Core Team, 2025). Separate meta-analyses were performed for the associations and regressions.

## 3. Results

### 3.1. Searched Articles

The flow of the article selection process for the systematic review and meta-analysis, showing the exclusion and inclusion criteria, is presented in Figure 2. The results indicated that a total of three hundred and six (*n* = 306) articles were found in the five databases used (Google Scholar, PubMed, Scopus, Web of Science and OpenAlex). A total of two hundred and seven (*n* = 207) articles remained after the removal of duplicates, and seventy-seven (*n* = 77) articles were screened for eligibility, resulting in forty-five (*n* = 45) articles being included in the study for meta-analysis.

### 3.2. Characterization of the Included Articles

Table 1 shows the characteristics of the forty-five (*n* = 45) included articles published from 2005 to 2025, with the sample size ranging from 30 to 1072 goats. The included studies investigated different goat breeds, age groups and sexes. This variation indicates that breed, age, and sex are important factors to consider when estimating body weight from linear body measurements.

### 3.3. Methodological Quality of Included Studies

The methodological quality of the 45 included publications was assessed using the 8-item JBI critical appraisal checklist. The findings showed that the quality scores ranged from 6 to 8 out of 8. All 45 studies were classified as having low overall risk. The detailed item-level assessments are presented in Supplementary Table S2.

### 3.4. Distribution of Studies by Year

**Figure 3:**
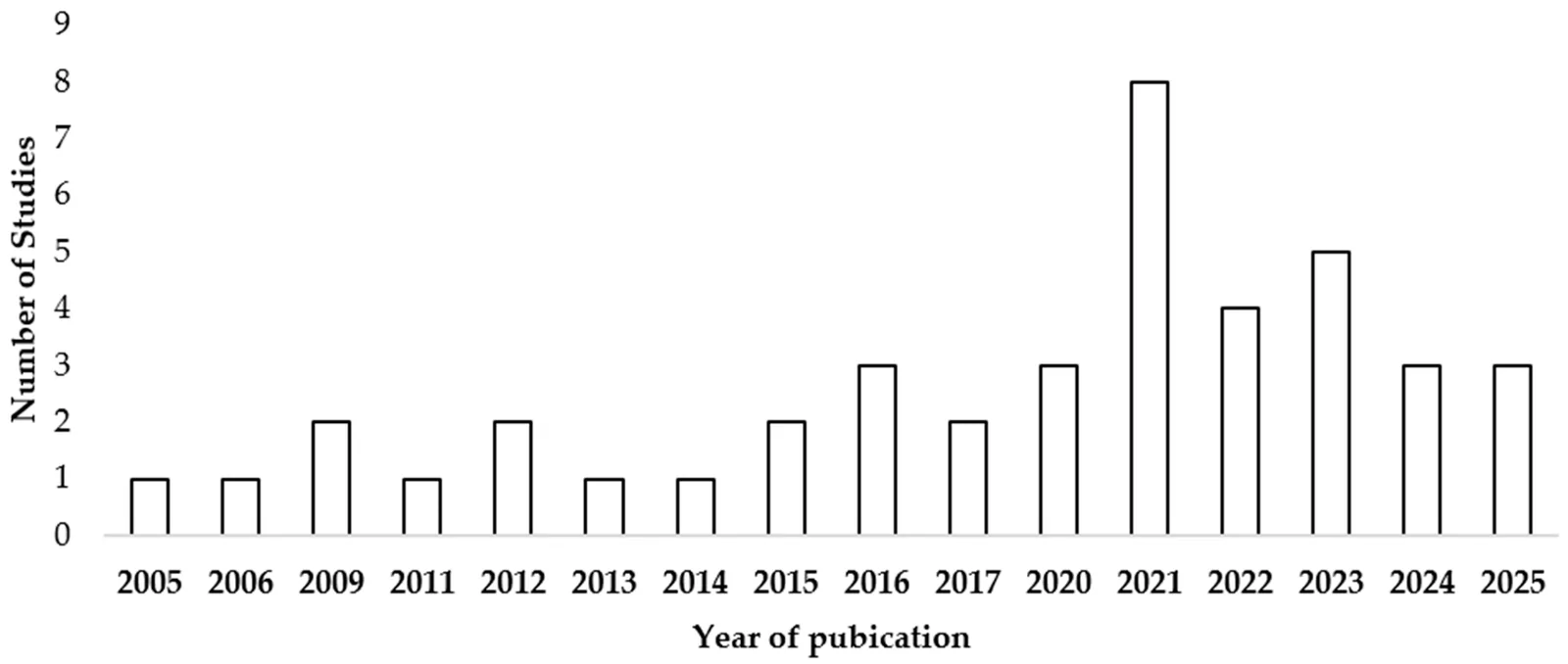
Publications by year.

Figure 3 presents the distribution of the included studies according to publication year. The studies were published between 2005 and 2025, with the most studies published in 2021 (8/45), followed by 2023 (5/45) and 2022 (4/45). The number of included studies was generally higher from 2020 onwards, indicating that a substantial proportion of the available evidence included in this study was published during the most recent years of the study period.

### 3.5. Distribution of Studies by Country

**Figure 4:**
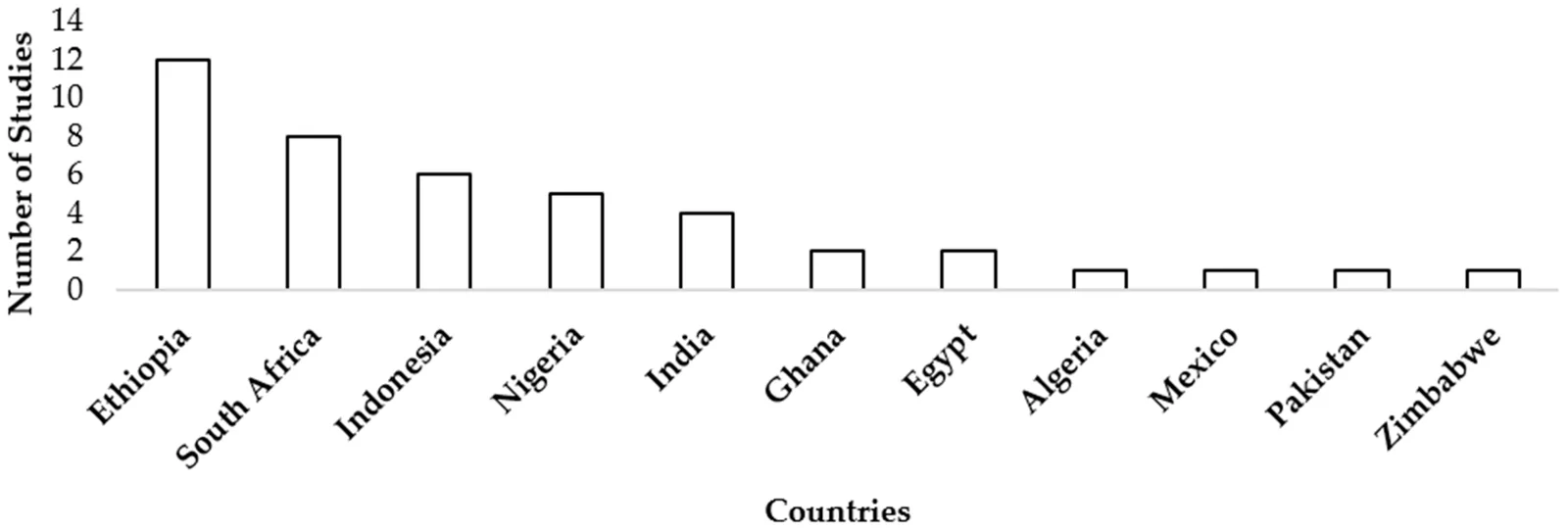
Publications by country.

The geographical distribution of the included studies is presented in Figure 4. The studies originated from eleven countries, with Ethiopia contributing the highest number of studies (12/45; 26.7%), followed by South Africa (8/45; 17.8%), Indonesia (6/45; 13.3%), Nigeria (5/45; 11.1%) and India (4/45; 8.9%). Ghana and Egypt each contributed two articles (2/45; 4.4%), whereas Algeria, Mexico, Pakistan and Zimbabwe each contributed one article (1/45; 2.2%). The included studies were geographically diverse, although the largest proportion of studies originated from Ethiopia and South Africa.

### 3.6. Distribution of Studies by Breed Type

The distribution of included studies according to breed type is presented in Table 2. The majority of the included studies (40/45; 88.9%) investigated indigenous goat breeds, whereas only a smaller number of included studies investigated crossbred goat breeds (2/45; 4.4%). The predominance of indigenous goat breeds indicates that the available evidence on the estimation of body weight using linear body measurements is largely derived from locally adapted goat populations.

### 3.7. Distribution of Included Studies for Meta-Analysis of Correlations

Table 3 presents the summary structure of the dataset used in the meta-analysis. For the heart girth and body length meta-analysis, 43 studies were included, with 30 of them contributing multiple effect sizes. For the withers height meta-analysis, 37 studies were included, with 27 of them contributing multiple effect sizes and 113 correlation coefficients. Therefore, a multilevel meta-analytic model was used to account for the dependence among effect sizes originating from the same study.

### 3.8. Meta-Analysis of Correlations

A multilevel random-effects model fitted using restricted maximum likelihood (REML) was used for meta-analysis of correlations between body weight and linear body measurements (Table 4) to account for the dependence among multiple effect sizes originating from the same study. A strong positive pooled correlation was observed between BW and HG (r = 0.83, 95% CI: 0.79–0.87), BW and BL (r = 0.79, 95% CI: 0.74–0.83), and BW and WH (r = 0.72, 95% CI: 0.64–0.78). The pooled correlations were statistically significant (*p* < 0.001), indicating a strong positive association between body weight and linear body measurements across the included studies.

### 3.9. Heterogeneity of Pooled Correlations

Table 5 shows the heterogeneity of pooled correlations between body weight and linear body measurements. Heterogeneity analysis revealed significant variation among the effect sizes in HG (Q = 9472.82, df = 128, *p* < 0.001), BL (Q = 7774.51, df = 125, *p* < 0.001), and WH (Q = 7916.60, df = 112, *p* < 0.001). The high I^2^ values observed in HG, BL and WH were 96.74%, 96.17% and 97.11%, respectively, indicating that variability in the observed effect sizes was attributable to differences between studies rather than sampling error. These findings demonstrate considerable variation in the strength of the association between body weight and linear body measurements among the included studies and support the use of a multilevel random-effects meta-analytic model.

### 3.10. Random-Effects Variance of Correlations

Table 6 shows the random-effects variance test. The likelihood-ratio test (LRT) was highly significant in HG (LRT = 6787.83, df = 1, *p* < 0.0001), BL (LRT = 4993.02, df = 1, *p* < 0.0001), and WH (LRT = 4983.10, df = 1, *p* < 0.0001), providing evidence that the estimated random-effects variances were different from zero. This supports the use of a multilevel random-effects model to account for between-study variability.

### 3.11. Prediction Intervals of Correlations

Table 7 presents the 95% prediction intervals for the correlations between body weight and linear body measurements. The 95% prediction intervals for the correlation between BW and HG, BW and BL, and BW and WH ranged from 0.33 to 0.97, 0.26 to 0.95, and 0.03 to 0.94, respectively, indicating that the true correlation in a future comparable study could vary across these ranges.

### 3.12. Forest Plots of Correlations

The forest plot demonstrated predominantly positive associations between body weight and heart girth (Supplementary Figure S1), body weight and body length (Supplementary Figure S2), and body weight and withers height (Supplementary Figure S3). The magnitude of the individual correlation coefficients varied from weak to very strong positive associations, with some studies having confidence intervals that included zero. The pooled correlation coefficients were 0.83 (95% CI: 0.79–0.87), 0.79 (95% CI: 0.74–0.83), and 0.72 (95% CI: 0.64–0.78) for HG, BL, and WH, respectively, indicating a strong positive association between body weight and linear body measurements.

### 3.13. Multilevel Small-Study Effects of Correlations

Table 8 presents the multilevel small-study effect tests. The small-study effect tests were not statistically significant for HG (*p* = 0.11) and WH (*p* = 0.20) but were statistically significant for BL (*p* = 0.03). The findings were non-significant for all the linear body measurements (HG, *p* = 0.14; BL, *p* = 0.18; WH, *p* = 0.30) after robust adjustment for dependence among effect sizes, indicating no evidence of a small-study effect in the association between body weight and linear body measurements.

### 3.14. Meta-Regression of Correlations

Table 9 shows the univariable and multivariable meta-regression analysis for the association between body weight and linear body measurements. Continent, age group, sex, breed, publication period, and sample size were used as moderators. The univariable meta-regression indicated that age significantly moderated the correlation between BW and HG (QM = 61.440, df = 2, *p* < 0.001), indicating that the strength of association between body weight and heart girth varies across age groups. For BL, age (QM = 52.87, df = 2, *p* < 0.001), sex (QM = 22.10, df = 2, *p* < 0.001) and sample size (QM = 14.99, df = 1, *p* < 0.001) were significant moderators, indicating that the strength of the relationship between body weight and body length varies across age groups, sexes and sample sizes. For WH, all the moderators were significant except publication period (QM = 3.15, df = 2, *p* = 0.207) and sample size (QM = 3.12, df = 1, *p* = 0.077), indicating that the strength of the correlation between body weight and withers height did not vary across publication periods and sample sizes but varied across continents, age groups, and breed types. In multivariable meta-regression analysis, moderators were statistically significant in all the linear body measurements, indicating that the magnitude of the association varied according to goat and study characteristics represented by the used moderators.

### 3.15. Leave-One-Publication-Out Sensitivity Analysis of Correlations

The leave-one-publication-out sensitivity analysis showed that the pooled correlation estimates remained relatively stable following the sequential exclusion of individual studies. In HG (Supplementary Table S3), the pooled correlation coefficients ranged from 0.83 to 0.84, compared with the overall pooled correlation of 0.83. In BL (Supplementary Table S4), the pooled correlation coefficients ranged from 0.78 to 0.79, compared with the overall pooled correlation of 0.79. In WH (Supplementary Table S5), the pooled correlation estimates ranged from 0.70 to 0.73, compared with the overall pooled correlation of 0.72. Overall, the relatively small changes in the pooled estimates after sequentially excluding individual studies indicate that no single study strongly influenced the overall estimated correlation between body weight and linear body measurements. Therefore, the meta-analysis results on the correlation between body weight and linear body measurements are not dependent on one study.

### 3.16. Distribution of Included Studies for Meta-Analysis of Regressions

The distribution of studies and regression coefficients included in the meta-analysis is presented in Table 10. The meta-analysis included 3 studies for HG, contributing 10 regression coefficients, and 4 studies for both BL and WH, contributing 11 and 15 regression coefficients, respectively. A total of 3 studies contributed multiple effect sizes for each of the three linear body measurements. The clustering of multiple effect sizes within studies was accounted for using a multilevel random-effects meta-analysis.

### 3.17. Pooled Regression Coefficients and 95% Prediction Intervals of Regressions

The pooled regression coefficient results for heart girth (HG), body length (BL), and withers height (WH) are presented in Table 11. The meta-analysis revealed variation in the strength and statistical significance of the pooled regression coefficients among the three linear body measurements. The results showed that HG, BL and WH had pooled regression coefficients of 0.65 (95% CI: 0.55–0.74), 0.40 (95% CI: 0.04–0.76), and 0.06 (95% CI: −0.02–0.14), respectively. The 95% prediction interval for HG remained entirely positive (95% CI: 0.55–0.74), while those for BL (95% CI: −0.23–1.04) and WH (95% CI: −0.02–0.14) included zero. Overall, the findings indicate that the magnitude and consistency of the regression relationship with body weight differed among the three linear body measurements.

### 3.18. Forest Plot of Regression Between Body Weight and Heart Girth

**Figure 5:**
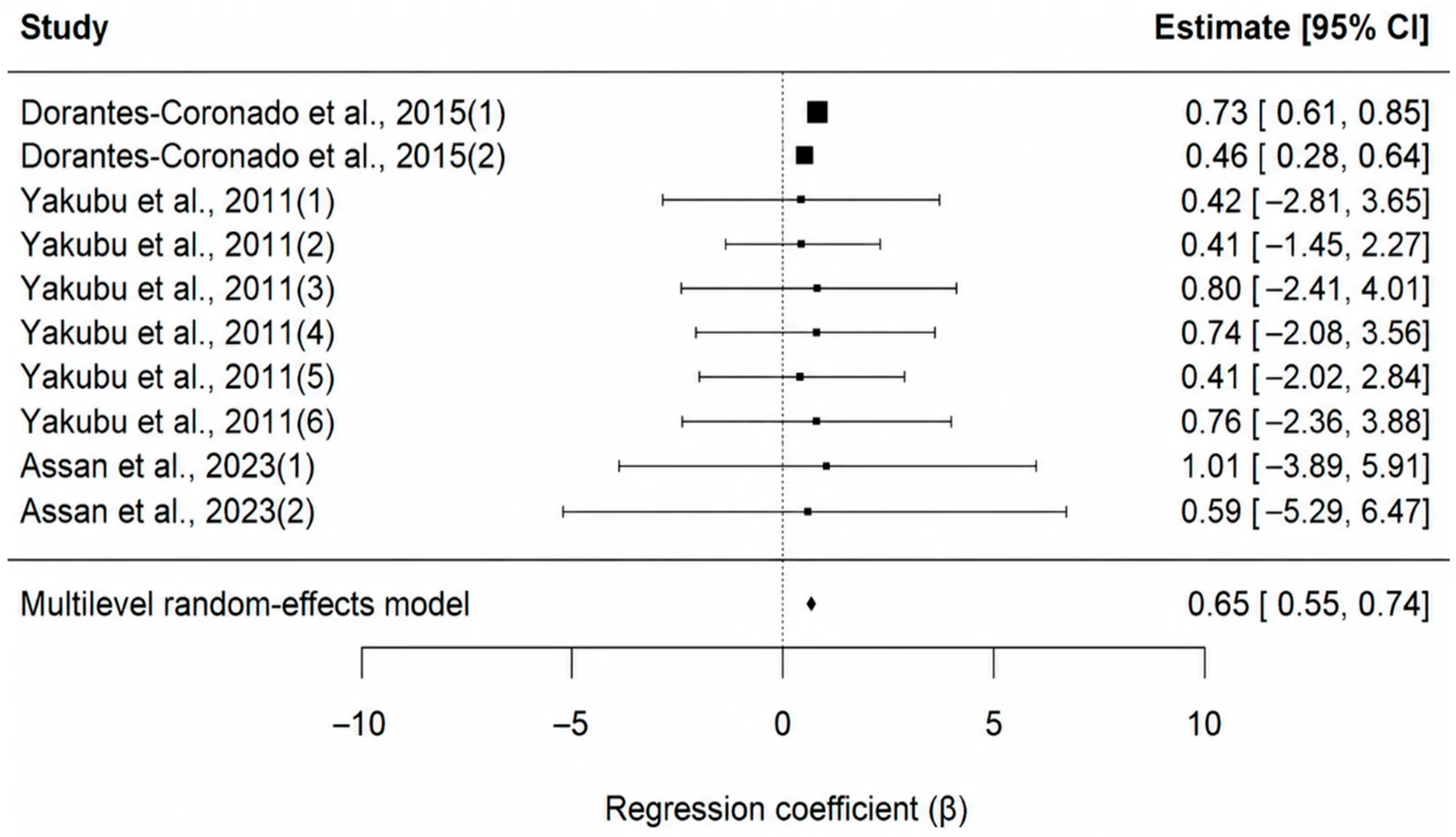
Forest plot of regression between body weight and heart girth. Dorantes-Coronado et al., 2025 [18], Yakubu et al., 2011 [12], Assan et al., 2023 [39]. Numbers in parentheses after the study citation denote distinct effect sizes extracted from the same study.

The forest plot showed variation in the regression coefficients between body weight and heart girth across 10 regression coefficients from the 3 studies (Figure 5). The individual estimates ranged from 0.41 to 1.01, with several estimates having wide 95% confidence intervals that included 0. The multilevel random-effects model yielded a pooled regression coefficient of 0.65 (95% CI: 0.55–0.74), indicating a positive overall association between body weight and heart girth.

### 3.19. Funnel Plot of Regression Between Body Weight and Heart Girth

**Figure 6:**
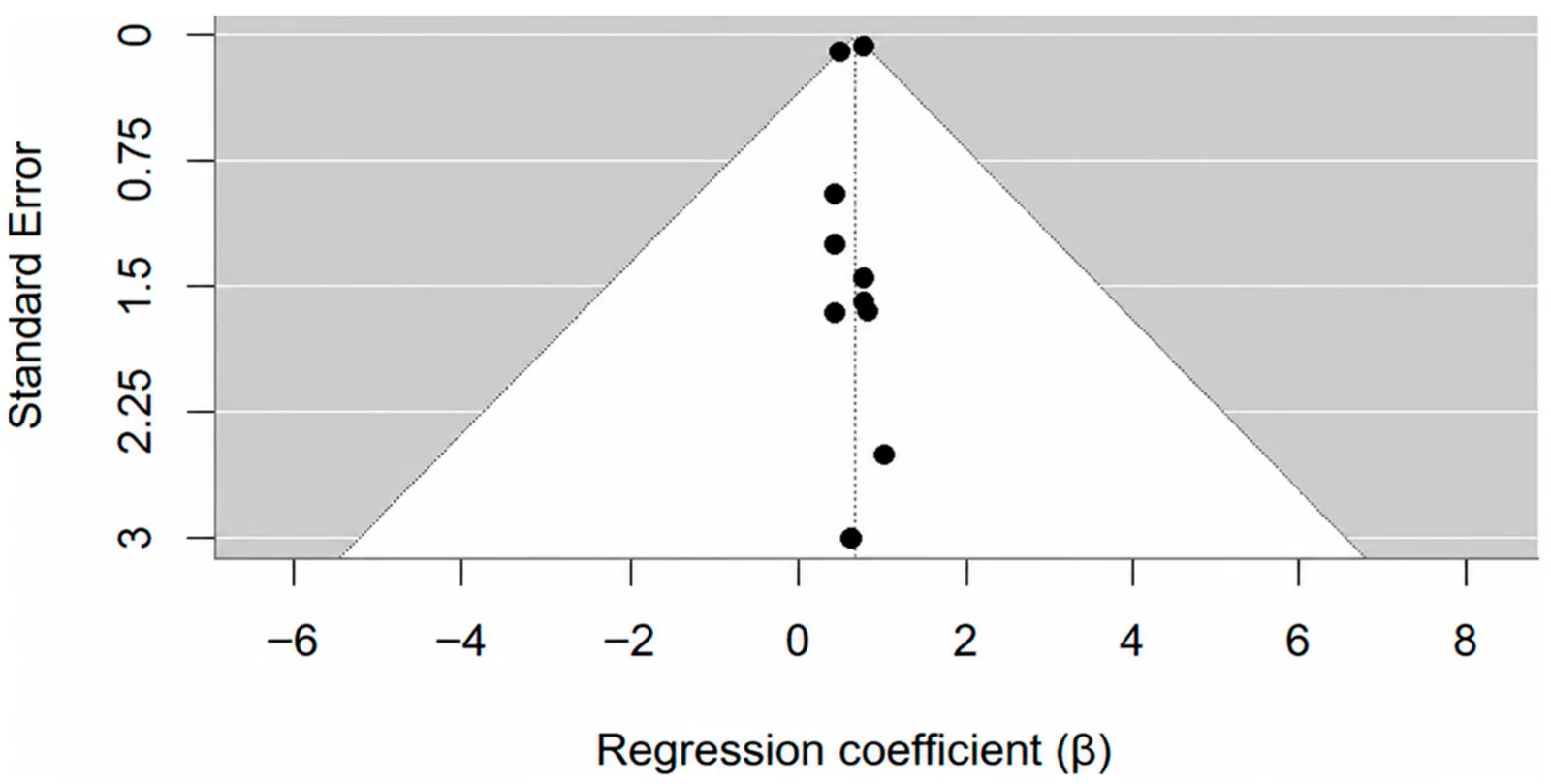
Funnel plot of regression coefficients between body weight and heart girth.

Figure 6 presents the funnel plot of regression coefficients between body weight and heart girth. The funnel plot shows that all 10 regression coefficients are generally distributed around the pooled estimate of approximately 0.65, with no clear visual evidence of substantial funnel-plot asymmetry.

### 3.20. Forest Plot of Regression Between Body Weight and Body Length

**Figure 7:**
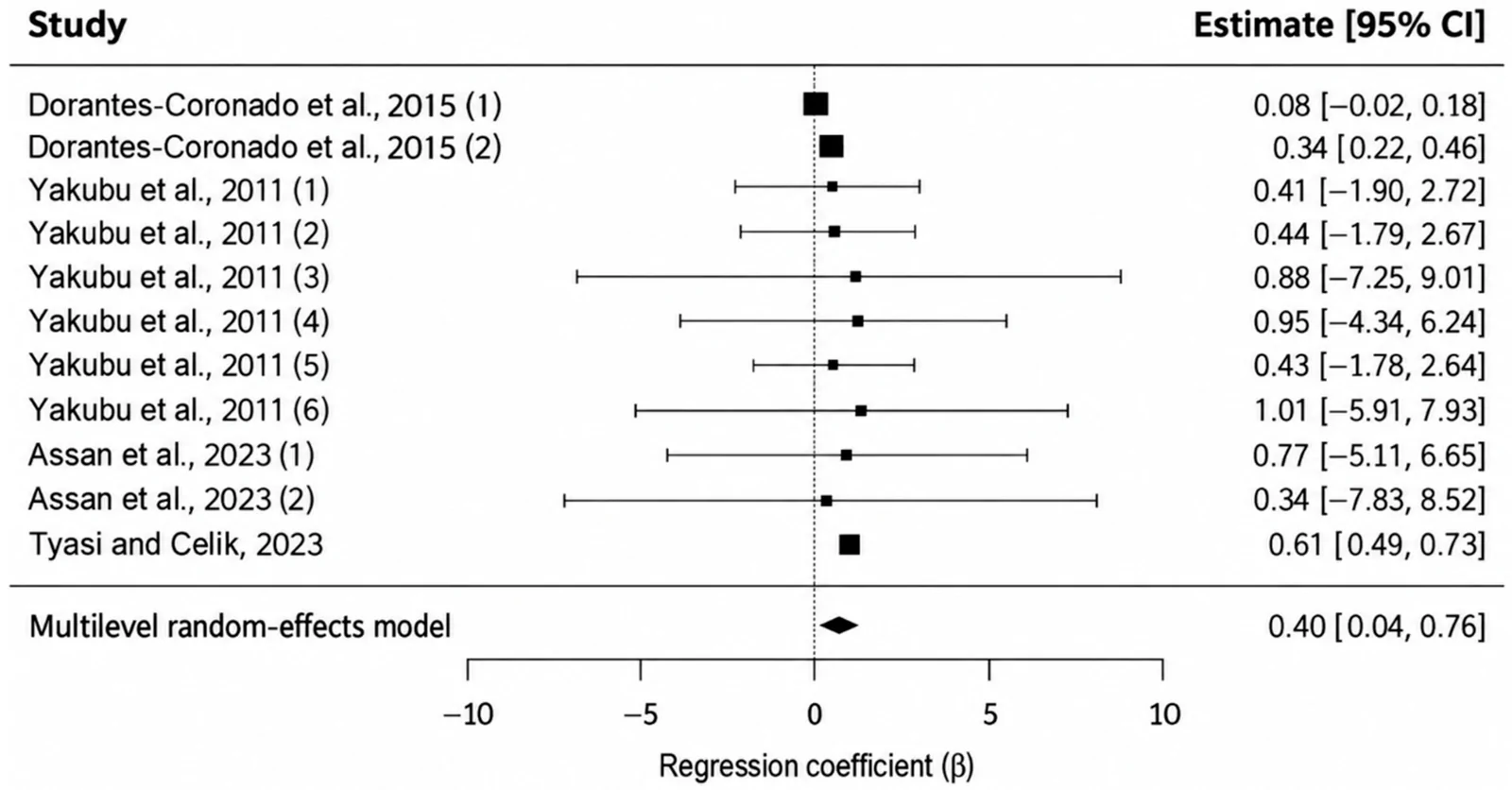
Forest plot of regression between body weight and body length. Dorantes-Coronado et al., 2025 [18], Yakubu et al., 2011 [12], Assan et al., 2023 [39], Tyasi and Celik, 2023 [42]. Numbers in parentheses after the study citation denote distinct effect sizes extracted from the same study.

The forest plot showed variation in the regression coefficients between body weight and body length across 11 regression coefficients from 4 studies (Figure 7). The individual estimates ranged from 0.08 to 1.01, with several estimates having wide 95% confidence intervals that included 0. The multilevel random-effects model yielded a pooled regression coefficient of 0.40 (95% CI: 0.04–0.76), indicating a positive overall association between body weight and body length.

### 3.21. Funnel Plot of Regression Between Body Weight and Body Length

**Figure 8:**
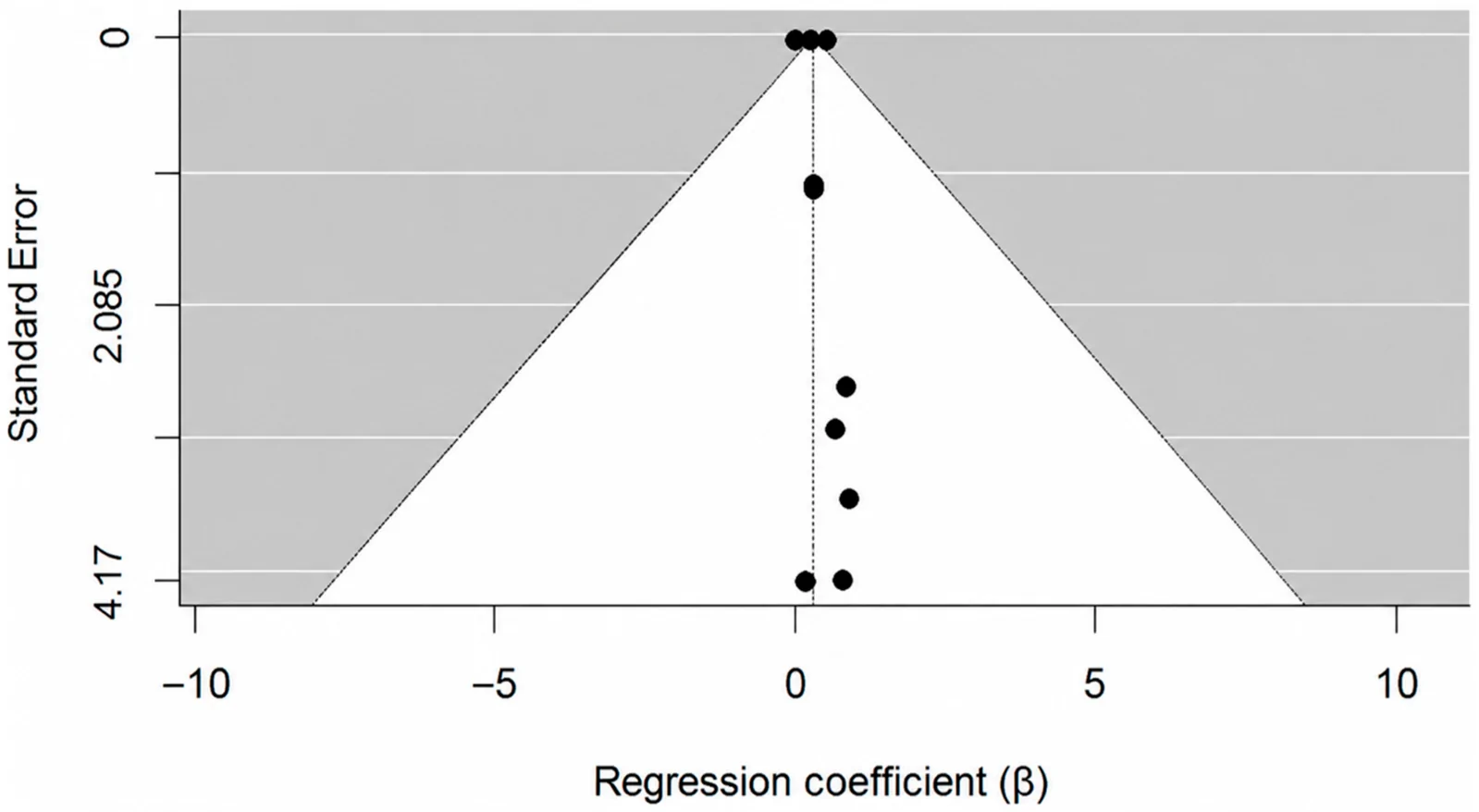
Funnel plot of regression coefficients between body weight and body length.

Figure 8 represents the funnel plot of the regression coefficients between body weight and body length. The funnel plot shows that the 11 regression coefficients are generally distributed around the pooled estimate of approximately 0.40, with no clear visual evidence of substantial funnel-plot asymmetry.

### 3.22. Forest Plot of Regression Between Body Weight and Withers Height

**Figure 9:**
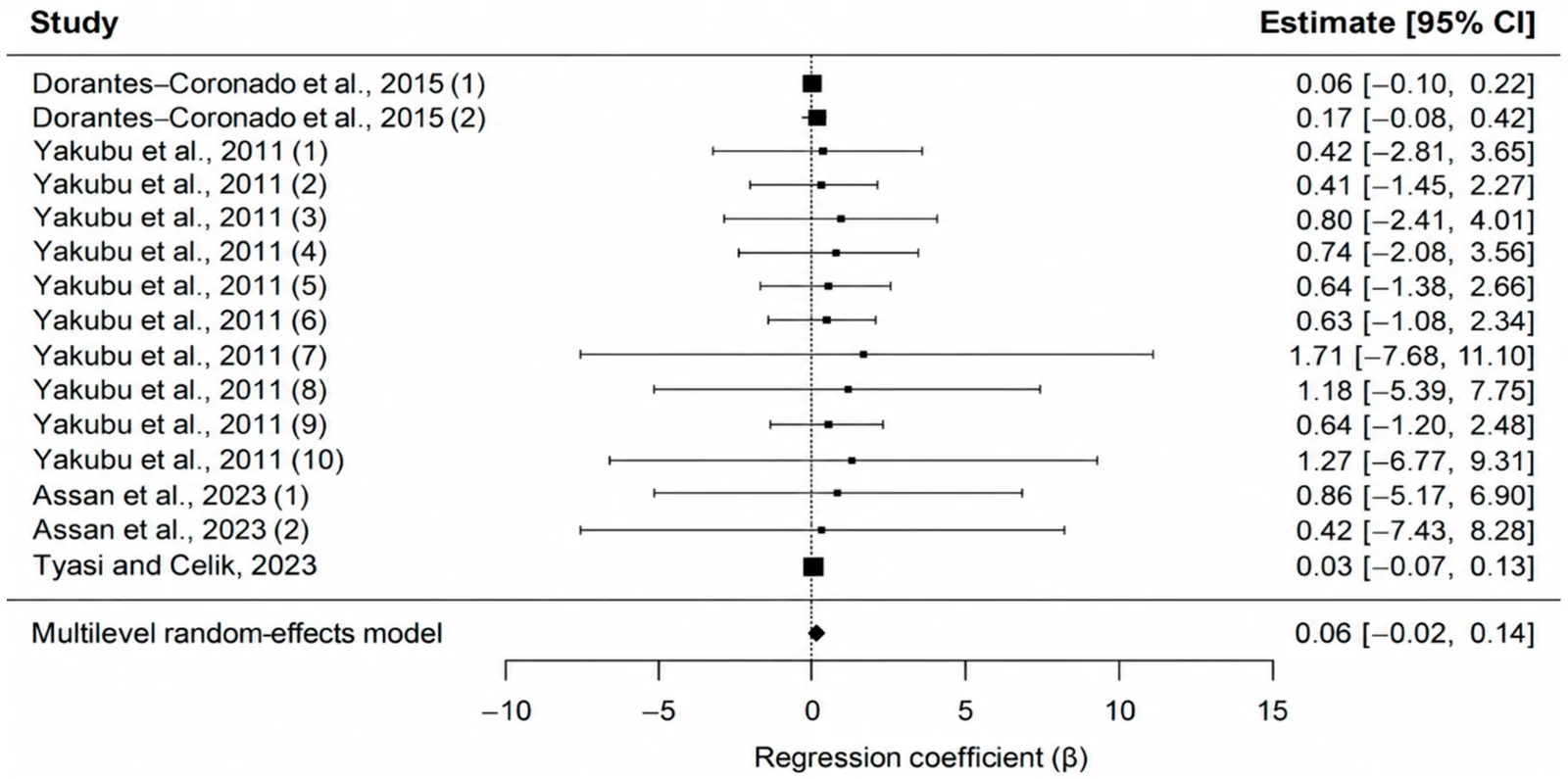
Forest plot of regression between body weight and withers height. Dorantes-Coronado et al., 2025 [18], Yakubu et al., 2011 [12], Assan et al., 2023 [39], Tyasi and Celik, 2023 [42]. Numbers in parentheses after the study citation denote distinct effect sizes extracted from the same study.

The forest plot showed variation in the regression coefficients between body weight and withers height across 15 regression coefficients from 4 studies (Figure 9). The individual estimates ranged from 0.03 to 1.71, with several estimates having wide 95% confidence intervals that included 0. The multilevel random-effects model yielded a pooled regression coefficient of 0.06 (95% CI: −0.02–0.14), indicating a positive but non-significant overall association between BW and WH.

### 3.23. Funnel Plot of Regression Between Body Weight and Withers Height

**Figure 10:**
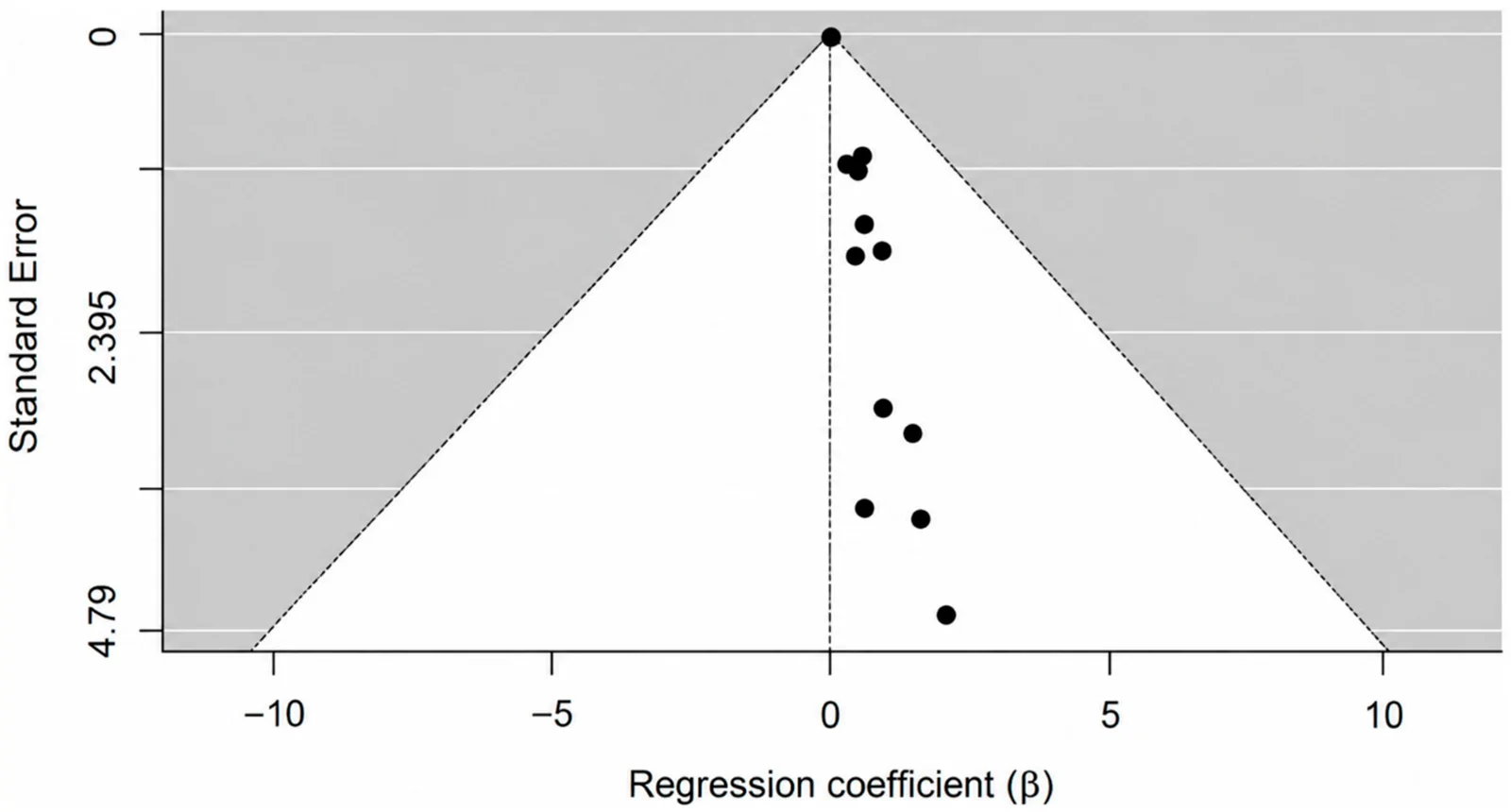
Funnel plot of regression coefficients between body weight and withers height.

Figure 10 represents the funnel plot of the regression coefficients between body weight and withers height. The funnel plot shows that the 15 regression coefficients are generally distributed around the pooled estimate of approximately 0.06, with most estimates concentrated close to the pooled regression coefficient. There is no clear visual evidence of substantial funnel-plot asymmetry, although the estimates with larger standard errors show greater dispersion.

## 4. Discussion

In animal production, live body weight is a very important factor playing a critical role in management [51,52]. Accurate prediction of body weight in animals is essential for proper animal management practices [52]. There is variation in the reported results on the impact of linear body measurements on live body weight of goats. Hence, this study aimed to use the meta-analysis approach to combine correlation and regression outcomes of published studies to provide precise outcomes that can be useful for animal husbandry. A multilevel random-effects model was employed for correlation and regression meta-analyses. Strong positive significant coverall correlations were observed between body weight and heart girth (r = 0.83, 95% CI: 0.79–0.87), body length (r = 0.79, 95% CI: 0.74–0.83), and withers height (r = 0.72, 95% CI: 0.64–0.78). These results provide evidence that heart girth, body length and withers height are among the important linear body measurements that need to be considered for estimating body weight in the absence of weighing equipment in goats. There are meta-analysis findings in other livestock species on the correlation between body weight and linear body measurements. In sheep, similar results [53] found a strong positive statistical correlation between live body weight and heart girth (r = 0.83, 95% CI: 0.80–0.85). In cattle, similar results [54] reported a strong positive statistical correlation between live body weight and heart girth (r = 0.88, 95% CI: 0.86–0.91). The strong positive association between body weight and heart girth might be because, as animals grow, expansion of the thoracic cavity accompanies development of the heart and lungs, while adipose tissue and muscle are deposited around the thorax area. Therefore, an increase in overall body mass is often accompanied by an increase in chest circumference. According to Mokoena et al. [2], live body weight and heart girth are influenced by similar genetic factors. This study showed high heterogeneity across studies (I^2^ > 96%), indicating substantial variability among the included studies. The meta-regression was conducted to fit continent, age, sex, breed, publication period and sample size. The study found that these factors contributed substantially to the diversity of results. Therefore, they might be considered when investigating the relationship between body weight and heart girth, body length, and withers height. Since many studies had multiple effect sizes, the leave-one-publication-out sensitivity analysis was performed by removing all effect sizes belonging to one study at a time, and the study suggests that the pooled estimates were robust, with no single study substantially altering the pooled correlation coefficients. This study also used a multilevel random-effects model analysis on regression results for prediction of live body weight from heart girth, body length and withers weight. The multilevel meta-analysis showed positive pooled regression coefficients for all three linear body measurements, although the statistical evidence differed among the measurements. For heart girth, the pooled regression coefficient was 0.65 kg/cm (95% CI: 0.55–0.74), with the confidence interval excluding zero. For body length, the pooled regression coefficient was 0.40 kg/cm (95% CI: 0.04–0.76), also indicating a positive association with body weight. In contrast, the pooled regression coefficient for withers height was 0.06 kg/cm (95% CI: −0.02–0.14), with the confidence interval including zero. The pooled regression estimates indicated positive associations between body weight and heart girth, body length, and withers height. The associations for heart girth and body length were statistically supported, as their 95% confidence intervals excluded zero, whereas the confidence interval for withers height included zero, indicating greater uncertainty regarding the association between withers height and body weight. Nakanish et al. [55] indicated that heart girth and body length are useful for the development of the equation to estimate live body weight of Japanese Sannen goats. The heart girth measurement provides a simple and low-cost method for estimating live body weight, particularly useful in resource-limited settings where weighing equipment is unavailable [3]. A major strength of this study lies in the inclusion of many datasets around different goat breeds coming from different geographical regions. However, several limitations should be acknowledged. The high heterogeneity among the studies shows the variability in many aspects of the studies. Furthermore, the limited number of studies that estimated live body weight using regression analysis may have reduced the precision of pooled estimates. To address these limitations, future research should focus on estimating live body weight from more linear body measurement traits.

## 5. Conclusions

The findings of this multilevel meta-analysis reveal positive associations between live body weight and heart girth, body length and withers height of goats. The pooled correlation analysis showed strong positive associations between body weight and heart girth, body length, and withers height, with all associations being statistically significant. The pooled regression analysis further showed positive regression estimates for heart girth, body length, and withers height. The regression estimates for heart girth and body length were statistically supported, whereas the confidence interval for withers height included zero, indicating greater uncertainty around its pooled regression estimate. These findings indicate that heart girth, body length, and withers height are useful phenotypic indicators associated with live body weight in goats. The results provide quantitative evidence supporting the use of linear body measurements for body weight estimation, particularly in production systems where access to weighing equipment may be limited. Further research involving larger and more diverse goat populations is recommended to improve the precision and applicability of live body weight prediction models across breeds, production systems, and environments.

## Figures and Tables

**Table 1:** Studies included.

Author	Year	Country	Breed	Sex	Age	N
Thiruvenkadan, 2005 [8]	2005	India	Kanniadu	M, F	A1	328
Khan et al., 2006 [9]	2006	Pakistan	Indian Beetal	M, F	A1, A2, A2, A3	86
Akpa et al., 2009 [10]	2009	Nigeria	Red Sokoto	M, F	A1	1000
Yakubu, 2009 [11]	2009	Nigeria	West African Dwarf	M, F	A1	58
Yakubu et al., 2011 [12]	2011	Nigeria	Non-descript	M, F	A1, A2	135
Raja et al., 2012 [13]	2012	India	Attappady Black	M, F	A1	824
Yakubu and Mohammed 2012 [14]	2012	Nigeria	Red Sokoto	M, F	A2	142
Abdel-Mageed and Ghanem 2013 [15]	2013	Egypt	Zaraibi, Damascus, Barki	M, F	A1	30
Tekle 2014 [16]	2014	Ethiopia	Afar	M, F	A1, A2, A3	318
Birteeb and Lomo 2015 [17]	2015	Ghana	West African Dwarf	M, F	A1, A2, A3	325
Dorantes-Coronado et al., 2015 [18]	2015	Mexico	Local	F	A3	318
Hadi et al., 2016 [19]	2016	Indonesia	Boerawa (Boer × Ettawa great)	M	A3	120
Hagos et al., 2016 [20]	2016	Ethiopia	Begait	M, F	A3	615
Sam et al., 2016 [21]	2016	Nigeria	West African Dwarf	M, F	A1, A2, A3	250
Gatew et al., 2017 [22]	2017	Ethiopia	Bati, Borana, Short-Eared Somali	M, F	A2	601
Zergaw et al., 2017 [23]	2017	Ethiopia	Central highland, Woyto-Guji	F	A3	531
Dakhlan et al., 2020 [24]	2020	Indonesia	Ettwa Great	F	A3	41
Karna et al., 2020 [25]	2020	India	Ganjam	M, F	A2, A3	1014
Mulai et al., 2020 [26]	2020	Ethiopia	Indigenous	M, F	A1, A2, A3	603
Dakhlan et al., 2021a [27]	2021	Indonesia	Saburai	F	A3	42
Dakhlan et al., 2021b [28]	2021	Indonesia	Saburai	M, F	A1	214
Mathapo and Tyasi 2021 [29]	2021	South Africa	Boer	M, F	A2	71
Ofori et al., 2021 [30]	2021	Ghana	West African Dwarf	M, F	A1, A2, A3	384
Ouchene-Khelifi and Ouchene 2021 [31]	2021	Algeria	Arabia, Makatia, Kabyle, Mzabite, Saneed, Alphine	M, F	A2, A3	1072
Rashijane et al., 2021 [32]	2021	South Africa	Boer	M, F	A2	72
Sheriff et al., 2021 [33]	2021	Ethiopia	Oromo	M, F	A1, A2, A3	747
Tyasi et al., 2021 [34]	2021	South Africa	Non-descript	M, F	A1, A2	83
Yousuf et al., 2021 [35]	2021	Ethiopia	Indigenous	M, F	A3	870
Getaneh et al., 2022 [36]	2022	Ethiopia	Non-descript	M, F	A2	706
Mokoena et al., 2022 [2]	2022	South Africa	Kalahari Red	M, F	A3	50
Muluneh and Tadesse 2022 [37]	2022	Ethiopia	Indigenous	M, F	A1, A2, A3	540
Negesse et al., 2022 [38]	2022	Ethiopia	Indigenous	M, F	A1, A2, A3	460
Assan et al., 2023 [39]	2023	Zimbabwe	Indigenous Matabele	F	A3	30
Dongre et al., 2023 [40]	2023	India	Udgiri	M, F	A1, A2	1070
Mallam et al., 2023 [41]	2023	Nigeria	Nigerian non-descript	M, F	A1	120
Rashijane et al., 2023 [3]	2023	South Africa	Savanah	M, F	A2, A3	173
Tyasi and Celik 2023 [42]	2023	South Africa	Boer	M, F	A1, A2, A3	400
Zewdie et al., 2023 [43]	2023	Ethiopia	Indigenous	M, F	A2, A3	630
Lendrawati et al., 2024 [44]	2024	Indonesia	Kacang	F	A1, A2, A3	91
Lestari et al., 2024 [45]	2024	Indonesia	Kacang	M, F	A2	209
Taye et el., 2024 [46]	2024	Ethiopia	Indigenous Highland	M, F	A1, A2, A3	550
Ahmed et al., 2025 [47]	2025	Egypt	Crossed Boer (Boer × Damascus)	F	A2, A3	40
Kegne et al., 2025 [48]	2025	Ethiopia	Indigenous	M, F	A2, A3	630
Shabangu et al., 2025 [49]	2025	South Africa	Savannah	M, F	A3	60
Tyasi, 2025 [50]	2025	South Africa	Non-descript	M, F	A1	100

M: Male, F: Female, A1: 0–12 Months, A2: >12–14 Months, A3: >24 months, N: number of goats.

**Table 2:** Distribution of studies by goat breed type.

Breed Type	Studies	No.
Indigenous	Thiruvenkadan (2005) [8]; Khan et al. (2006) [9]; Akpa et al. (2009) [10]; Yakubu (2009) [11]; Yakubu et al. (2011) [12]; Raja et al. (2012) [13]; Yakubu and Mohammed (2012) [14]; Abdel-Mageed and Ghanem (2013) [15]; Tekle (2014) [16]; Birteeb and Lomo (2015) [17]; Dorantes-Coronado et al. (2015) [18]; Hagos (2016) [20]; Sam et al. (2016) [21]; Gatew et al. (2017) [22]; Zergaw et al. (2017) [23]; Dakhlan et al. (2020) [24]; Karna et al. (2020) [25]; Mulai et al. (2020) [26]; Dakhlan et al. (2021a) [27]; Dakhlan et al. (2021b) [28]; Ofori et al. (2021) [30]; Ouchene-Khelifi and Ouchene (2021) [31]; Sheriff et al. (2021) [33]; Tyasi et al. (2021) [34]; Yousuf et al. (2021) [35]; Getaneh et al. (2022) [36]; Mokoena et al. (2022) [2]; Muluneh and Tadesse (2022) [37]; Negesse et al. (2022) [38]; Assan et al. (2023) [39]; Dongre et al. (2023) [40]; Mallam et al. (2023) [41]; Rashijane et al. (2023) [3]; Zewdie et al. (2023) [43]; Lendrawati et al. (2024) [44]; Lestari et al. (2024) [45]; Taye et al. (2024) [46]; Kegne et al. (2025) [48]; Shabangu et al. (2025) [49]; Tyasi (2025) [50]	40
Crossbred	Hadi et al. (2016) [19]; Ahmed et al. (2025) [47]	2
Commercial	Mathapo and Tyasi (2021) [29]; Rashijane et al. (2021) [32]; Tyasi and Celik (2023) [42]	3

**Table 3:** Summary structure of the dataset used for meta-analysis.

Characteristics	Heart Girth	Body Length	Withers Height
Number of studies	43	43	37
Number of correlation coefficients	129	126	113
Number of studies contributing multiple effect sizes	30	30	27

**Table 4:** Meta-analysis of correlations between body weight and linear body measurements.

Meta-Analysis Parameters	Heart Girth	Body Length	Withers Height
Number of correlation coefficients (k)	129	126	113
Pooled correlation coefficient (r)	0.83	0.79	0.72
95% confidence interval (CI)	0.79–0.87	0.74–0.83	0.64–0.78
Standard error	0.07	0.06	0.07
Test statistic (*z*)	17.84	17.11	12.34
*p*-value	<0.001	<0.001	<0.001

**Table 5:** Heterogeneity of correlations between body weight and linear body measurements.

Heterogeneity Parameters	Heart Girth	Body Length	Withers Height
Cochran’s Q	9472.82	7774.51	7916.60
Degrees of freedom (df)	128	125	112
*p*-value	<0.0001	<0.0001	<0.0001
Between-study variance (τ^2^)	0.19	0.16	0.19
Between-study SD (τ)	0.44	0.40	0.44
I^2^ (%)	96.74	96.17	97.11

SD: standard deviation.

**Table 6:** Test of the random-effects variance for correlations between body weight and linear measurements in goats.

Meta-Analysis Parameters	Heart Girth	Body Length	Withers Height
Likelihood-ratio test (LRT)	6787.83	4993.02	4983.10
Degrees of freedom (df)	1	1	1
*p*-value	<0.0001	<0.0001	<0.0001

**Table 7:** Prediction intervals for correlations between body weight and morphometric measurements.

Parameters	Heart Girth	Body Length	Withers Height
Pooled correlation coefficient (r)	0.834	0.79	0.72
95% prediction interval	0.33–0.97	0.26–0.95	0.03–0.94

**Table 8:** Small-study effect tests for the associations.

Parameters	Heart Girth	Body Length	Withers Height
Multilevel small-study effect test, *p*-value	0.11	0.03	0.20
Robust small-study effect test, *p*-value	0.14	0.18	0.30

**Table 9:** Meta-regression of the correlation between body weight and linear body measurements.

Moderators	Heart Girth QM (df)	*p*-Value	Body Length QM (df)	*p*-Value	Withers Height QM (df)	*p*-Value
Continent	0.28 (2)	0.87	1.59 (2)	0.45	12.43 (2)	0.002
Age	61.44 (2)	<0.001	52.87 (2)	<0.001	145.25 (2)	<0.001
Sex	3.58 (2)	0.17	22.10 (2)	<0.001	36.09 (2)	<0.001
Breed	3.25 (2)	0.197	2.62 (2)	0.27	13.12 (2)	0.001
Publication period	0.66 (2)	0.719	0.89 (2)	0.64	3.15 (2)	0.207
Sample size	3.44 (1)	0.064	14.99 (1)	<0.001	3.12 (1)	0.077
Multivariable meta-regression	73.87 (11)	<0.001	106.66 (11)	<0.001	201.05 (11)	<0.001
Multivariable model: Continent + Age + Sex + Breed + Publication Period + Sample Size						

**Table 10:** Summary of studies included in the regression meta-analysis.

Characteristics	Heart Girth	Body Length	Withers Height
Number of studies	3	4	4
Number of regression coefficients	10	11	15
Number of studies contributing multiple effect sizes	3	3	3

**Table 11:** Pooled regression coefficients and 95% prediction intervals.

Parameters	Heart Girth	Body Length	Withers Height
Pooled regression coefficient (β)	0.65	0.40	0.06
Confidence interval (CI)	0.55–0.74	0.04–0.76	−0.02–0.14
95% prediction interval	0.55–0.74	−0.23–1.04	−0.02–0.14
Cochran’s Q statistic (QE)	6.39	46.58	3.20
Degrees of freedom (df)	9	10	14
Study-level variance (τ^2^)	0.00	0.07	0.00
Heterogeneity *p*-value	0.70	<0.0001	0.9987

## Data Availability

No new data was created in this study. All data supporting the findings are publicly available in the original publications referenced in the manuscript.

## Supplementary Materials

The following supporting information can be downloaded at: https://www.mdpi.com/article/10.3390/ani16193107/s1, Figure S1: Forest plot of correlation between body weight and heart girth; Figure S2: Forest plot of correlation between body weight and body length; Figure S3: Forest plot of correlation between body weight and withers height; Table S1: Search strings and syntax used for database querying; Table S2: JBI critical appraisal of the methodological quality of the studies; Table S3: Leave-one-publication-out sensitivity analysis for the correlation between body weight and heart girth; Table S4: Leave-one-publication-out sensitivity analysis for the correlation between body weight and body length; Table S5: Leave-one-publication-out sensitivity analysis for the correlation between body weight and withers height.
