# Supplementary material for: Relationship Between Live Body Weight and Linear Body Measurements in Goats: A Meta-Analysis Using a Multilevel Random-Effects Model
Source: Animals (Basel). 2026 Oct 2;16(19):3107. doi: 10.3390/ani16193107 (PMC13663463; doi:10.3390/ani16193107)
Supplement: Supplementary file 1 [file animals-16-03107-s001.zip › Figure S1.pdf]

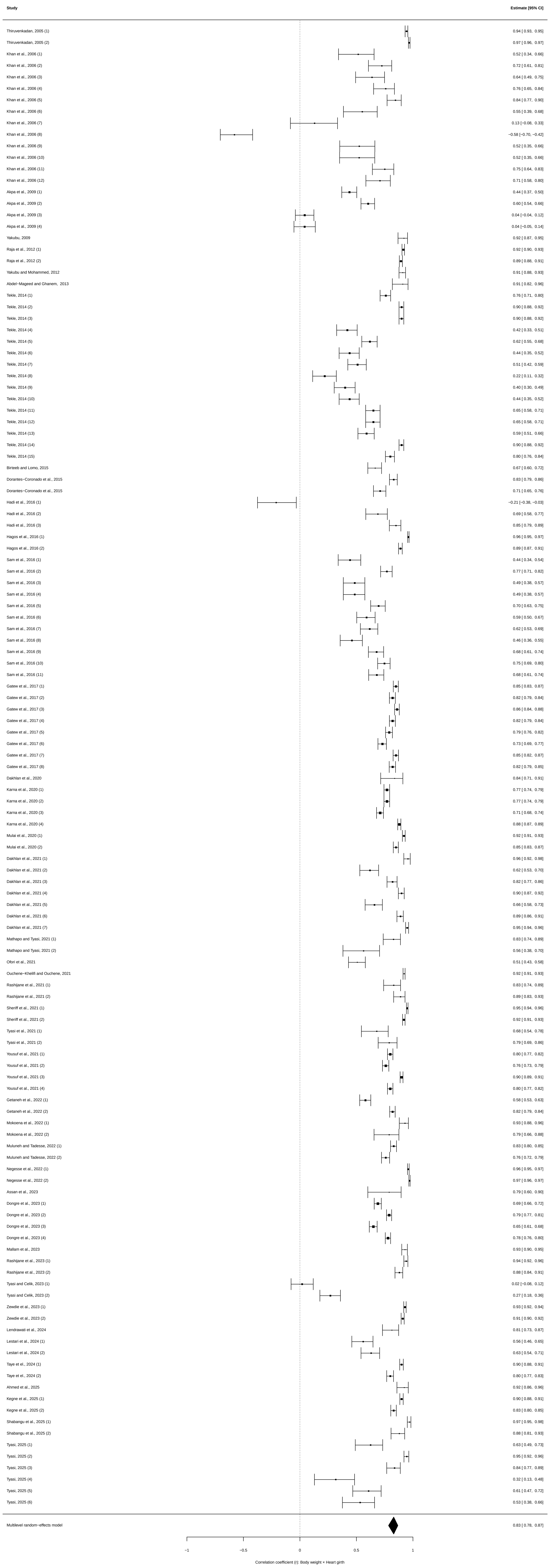

Study
Estimate [95% CI]
Thiruvenkadan, 2005 (1) 0.94 [0.93, 0.95]
Thiruvenkadan, 2005 (2) 0.97 [0.96, 0.97]
Khan et al., 2006 (1) 0.52 [0.34, 0.66]
Khan et al., 2006 (2) 0.72 [0.61, 0.81]
Khan et al., 2006 (3) 0.64 [0.49, 0.75]
Khan et al., 2006 (4) 0.76 [0.65, 0.84]
Khan et al., 2006 (5) 0.84 [0.77, 0.90]
Khan et al., 2006 (6) 0.55 [0.39, 0.68]
Khan et al., 2006 (7) 0.13 [−0.08, 0.33]
Khan et al., 2006 (8) −0.58 [−0.70, −0.42]
Khan et al., 2006 (9) 0.52 [0.35, 0.66]
Khan et al., 2006 (10) 0.52 [0.35, 0.66]
Khan et al., 2006 (11) 0.75 [0.64, 0.83]
Khan et al., 2006 (12) 0.71 [0.58, 0.80]
Akpa et al., 2009 (1) 0.44 [0.37, 0.50]
Akpa et al., 2009 (2) 0.60 [0.54, 0.66]
Akpa et al., 2009 (3) 0.04 [−0.04, 0.12]
Akpa et al., 2009 (4) 0.04 [−0.05, 0.14]
Yakubu, 2009 0.92 [0.87, 0.95]
Raja et al., 2012 (1) 0.92 [0.90, 0.93]
Raja et al., 2012 (2) 0.89 [0.88, 0.91]
Yakubu and Mohammed, 2012 0.91 [0.88, 0.93]
Abdel−Mageed and Ghanem, 2013 0.91 [0.82, 0.96]
Tekle, 2014 (1) 0.76 [0.71, 0.80]
Tekle, 2014 (2) 0.90 [0.88, 0.92]
Tekle, 2014 (3) 0.90 [0.88, 0.92]
Tekle, 2014 (4) 0.42 [0.33, 0.51]
Tekle, 2014 (5) 0.62 [0.55, 0.68]
Tekle, 2014 (6) 0.44 [0.35, 0.52]
Tekle, 2014 (7) 0.51 [0.42, 0.59]
Tekle, 2014 (8) 0.22 [0.11, 0.32]
Tekle, 2014 (9) 0.40 [0.30, 0.49]
Tekle, 2014 (10) 0.44 [0.35, 0.52]
Tekle, 2014 (11) 0.65 [0.58, 0.71]
Tekle, 2014 (12) 0.65 [0.58, 0.71]
Tekle, 2014 (13) 0.59 [0.51, 0.66]
Tekle, 2014 (14) 0.90 [0.88, 0.92]
Tekle, 2014 (15) 0.80 [0.76, 0.84]
Birteeb and Lomo, 2015 0.67 [0.60, 0.72]
Dorantes−Coronado et al., 2015 0.83 [0.79, 0.86]
Dorantes−Coronado et al., 2015 0.71 [0.65, 0.76]
Hadi et al., 2016 (1) −0.21 [−0.38, −0.03]
Hadi et al., 2016 (2) 0.69 [0.58, 0.77]
Hadi et al., 2016 (3) 0.85 [0.79, 0.89]
Hagos et al., 2016 (1) 0.96 [0.95, 0.97]
Hagos et al., 2016 (2) 0.89 [0.87, 0.91]
Sam et al., 2016 (1) 0.44 [0.34, 0.54]
Sam et al., 2016 (2) 0.77 [0.71, 0.82]
Sam et al., 2016 (3) 0.49 [0.38, 0.57]
Sam et al., 2016 (4) 0.49 [0.38, 0.57]
Sam et al., 2016 (5) 0.70 [0.63, 0.75]
Sam et al., 2016 (6) 0.59 [0.50, 0.67]
Sam et al., 2016 (7) 0.62 [0.53, 0.69]
Sam et al., 2016 (8) 0.46 [0.36, 0.55]
Sam et al., 2016 (9) 0.68 [0.61, 0.74]
Sam et al., 2016 (10) 0.75 [0.69, 0.80]
Sam et al., 2016 (11) 0.68 [0.61, 0.74]
Gatew et al., 2017 (1) 0.85 [0.83, 0.87]
Gatew et al., 2017 (2) 0.82 [0.79, 0.84]
Gatew et al., 2017 (3) 0.86 [0.84, 0.88]
Gatew et al., 2017 (4) 0.82 [0.79, 0.84]
Gatew et al., 2017 (5) 0.79 [0.76, 0.82]
Gatew et al., 2017 (6) 0.73 [0.69, 0.77]
Gatew et al., 2017 (7) 0.85 [0.82, 0.87]
Gatew et al., 2017 (8) 0.82 [0.79, 0.85]
Dakhlan et al., 2020 0.84 [0.71, 0.91]
Karna et al., 2020 (1) 0.77 [0.74, 0.79]
Karna et al., 2020 (2) 0.77 [0.74, 0.79]
Karna et al., 2020 (3) 0.71 [0.68, 0.74]
Karna et al., 2020 (4) 0.88 [0.87, 0.89]
Mulai et al., 2020 (1) 0.92 [0.91, 0.93]
Mulai et al., 2020 (2) 0.85 [0.83, 0.87]
Dakhlan et al., 2021 (1) 0.96 [0.92, 0.98]
Dakhlan et al., 2021 (2) 0.62 [0.53, 0.70]
Dakhlan et al., 2021 (3) 0.82 [0.77, 0.86]
Dakhlan et al., 2021 (4) 0.90 [0.87, 0.92]
Dakhlan et al., 2021 (5) 0.66 [0.58, 0.73]
Dakhlan et al., 2021 (6) 0.89 [0.86, 0.91]
Dakhlan et al., 2021 (7) 0.95 [0.94, 0.96]
Mathapo and Tyasi, 2021 (1) 0.83 [0.74, 0.89]
Mathapo and Tyasi, 2021 (2) 0.56 [0.38, 0.70]
Ofori et al., 2021 0.51 [0.43, 0.58]
Ouchene−Khelifi and Ouchene, 2021 0.92 [0.91, 0.93]
Rashijane et al., 2021 (1) 0.83 [0.74, 0.89]
Rashijane et al., 2021 (2) 0.89 [0.83, 0.93]
Sheriff et al., 2021 (1) 0.95 [0.94, 0.96]
Sheriff et al., 2021 (2) 0.92 [0.91, 0.93]
Tyasi et al., 2021 (1) 0.68 [0.54, 0.78]
Tyasi et al., 2021 (2) 0.79 [0.69, 0.86]
Yousuf et al., 2021 (1) 0.80 [0.77, 0.82]
Yousuf et al., 2021 (2) 0.76 [0.73, 0.79]
Yousuf et al., 2021 (3) 0.90 [0.89, 0.91]
Yousuf et al., 2021 (4) 0.80 [0.77, 0.82]
Getaneh et al., 2022 (1) 0.58 [0.53, 0.63]
Getaneh et al., 2022 (2) 0.82 [0.79, 0.84]
Mokoena et al., 2022 (1) 0.93 [0.88, 0.96]
Mokoena et al., 2022 (2) 0.79 [0.66, 0.88]
Muluneh and Tadesse, 2022 (1) 0.83 [0.80, 0.85]
Muluneh and Tadesse, 2022 (2) 0.76 [0.72, 0.79]
Negesse et al., 2022 (1) 0.96 [0.95, 0.97]
Negesse et al., 2022 (2) 0.97 [0.96, 0.97]
Assan et al., 2023 0.79 [0.60, 0.90]
Dongre et al., 2023 (1) 0.69 [0.66, 0.72]
Dongre et al., 2023 (2) 0.79 [0.77, 0.81]
Dongre et al., 2023 (3) 0.65 [0.61, 0.68]
Dongre et al., 2023 (4) 0.78 [0.76, 0.80]
Mallam et al., 2023 0.93 [0.90, 0.95]
Rashijane et al., 2023 (1) 0.94 [0.92, 0.96]
Rashijane et al., 2023 (2) 0.88 [0.84, 0.91]
Tyasi and Celik, 2023 (1) 0.02 [−0.08, 0.12]
Tyasi and Celik, 2023 (2) 0.27 [0.18, 0.36]
Zewdie et al., 2023 (1) 0.93 [0.92, 0.94]
Zewdie et al., 2023 (2) 0.91 [0.90, 0.92]
Lendrawati et al., 2024 0.81 [0.73, 0.87]
Lestari et al., 2024 (1) 0.56 [0.46, 0.65]
Lestari et al., 2024 (2) 0.63 [0.54, 0.71]
Taye et al., 2024 (1) 0.90 [0.88, 0.91]
Taye et al., 2024 (2) 0.80 [0.77, 0.83]
Ahmed et al., 2025 0.92 [0.86, 0.96]
Kegne et al., 2025 (1) 0.90 [0.88, 0.91]
Kegne et al., 2025 (2) 0.83 [0.80, 0.85]
Shabangu et al., 2025 (1) 0.97 [0.95, 0.98]
Shabangu et al., 2025 (2) 0.88 [0.81, 0.93]
Tyasi, 2025 (1) 0.63 [0.49, 0.73]
Tyasi, 2025 (2) 0.95 [0.92, 0.96]
Tyasi, 2025 (3) 0.84 [0.77, 0.89]
Tyasi, 2025 (4) 0.32 [0.13, 0.48]
Tyasi, 2025 (5) 0.61 [0.47, 0.72]
Tyasi, 2025 (6) 0.53 [0.38, 0.66]
Multilevel random−effects model 0.83 [0.78, 0.87]
−1
−0.5
0
0.5
1
Correlation coefficient (r): Body weight × Heart girth
