# Supplementary material for: Relationship Between Live Body Weight and Linear Body Measurements in Goats: A Meta-Analysis Using a Multilevel Random-Effects Model
Source: Animals (Basel). 2026 Oct 2;16(19):3107. doi: 10.3390/ani16193107 (PMC13663463; doi:10.3390/ani16193107)
Supplement: Supplementary file 1 [file animals-16-03107-s001.zip › Figure S2.pdf]

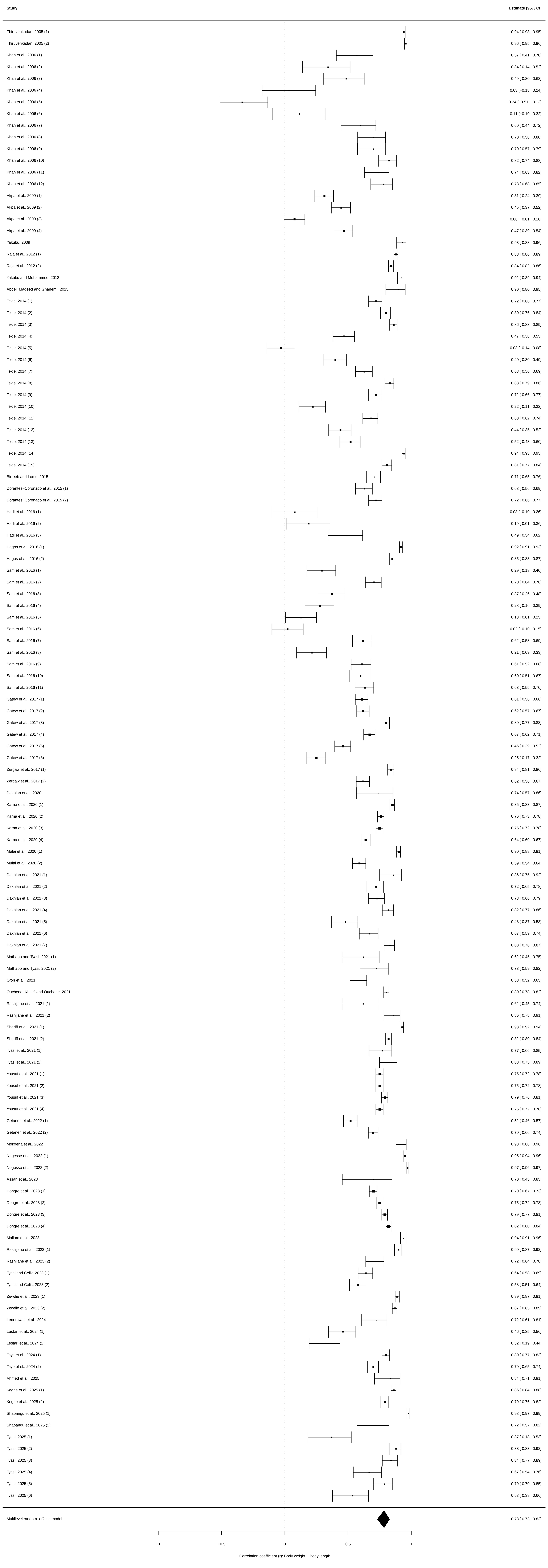
Study
Estimate [95% CI]
Thiruvenkadan. 2005 (1) 0.94 [0.93, 0.95]
Thiruvenkadan. 2005 (2) 0.96 [0.95, 0.96]
Khan et al.. 2006 (1) 0.57 [0.41, 0.70]
Khan et al.. 2006 (2) 0.34 [0.14, 0.52]
Khan et al.. 2006 (3) 0.49 [0.30, 0.63]
Khan et al.. 2006 (4) 0.03 [−0.18, 0.24]
Khan et al.. 2006 (5) −0.34 [−0.51, −0.13]
Khan et al.. 2006 (6) 0.11 [−0.10, 0.32]
Khan et al.. 2006 (7) 0.60 [0.44, 0.72]
Khan et al.. 2006 (8) 0.70 [0.58, 0.80]
Khan et al.. 2006 (9) 0.70 [0.57, 0.79]
Khan et al.. 2006 (10) 0.82 [0.74, 0.88]
Khan et al.. 2006 (11) 0.74 [0.63, 0.82]
Khan et al.. 2006 (12) 0.78 [0.68, 0.85]
Akpa et al.. 2009 (1) 0.31 [0.24, 0.39]
Akpa et al.. 2009 (2) 0.45 [0.37, 0.52]
Akpa et al.. 2009 (3) 0.08 [−0.01, 0.16]
Akpa et al.. 2009 (4) 0.47 [0.39, 0.54]
Yakubu, 2009 0.93 [0.88, 0.96]
Raja et al.. 2012 (1) 0.88 [0.86, 0.89]
Raja et al.. 2012 (2) 0.84 [0.82, 0.86]
Yakubu and Mohammed. 2012 0.92 [0.89, 0.94]
Abdel−Mageed and Ghanem. 2013 0.90 [0.80, 0.95]
Tekle. 2014 (1) 0.72 [0.66, 0.77]
Tekle. 2014 (2) 0.80 [0.76, 0.84]
Tekle. 2014 (3) 0.86 [0.83, 0.89]
Tekle. 2014 (4) 0.47 [0.38, 0.55]
Tekle. 2014 (5) −0.03 [−0.14, 0.08]
Tekle. 2014 (6) 0.40 [0.30, 0.49]
Tekle. 2014 (7) 0.63 [0.56, 0.69]
Tekle. 2014 (8) 0.83 [0.79, 0.86]
Tekle. 2014 (9) 0.72 [0.66, 0.77]
Tekle. 2014 (10) 0.22 [0.11, 0.32]
Tekle. 2014 (11) 0.68 [0.62, 0.74]
Tekle. 2014 (12) 0.44 [0.35, 0.52]
Tekle. 2014 (13) 0.52 [0.43, 0.60]
Tekle. 2014 (14) 0.94 [0.93, 0.95]
Tekle. 2014 (15) 0.81 [0.77, 0.84]
Birteeb and Lomo. 2015 0.71 [0.65, 0.76]
Dorantes−Coronado et al.. 2015 (1) 0.63 [0.56, 0.69]
Dorantes−Coronado et al.. 2015 (2) 0.72 [0.66, 0.77]
Hadi et al.. 2016 (1) 0.08 [−0.10, 0.26]
Hadi et al.. 2016 (2) 0.19 [0.01, 0.36]
Hadi et al.. 2016 (3) 0.49 [0.34, 0.62]
Hagos et al.. 2016 (1) 0.92 [0.91, 0.93]
Hagos et al.. 2016 (2) 0.85 [0.83, 0.87]
Sam et al.. 2016 (1) 0.29 [0.18, 0.40]
Sam et al.. 2016 (2) 0.70 [0.64, 0.76]
Sam et al.. 2016 (3) 0.37 [0.26, 0.48]
Sam et al.. 2016 (4) 0.28 [0.16, 0.39]
Sam et al.. 2016 (5) 0.13 [0.01, 0.25]
Sam et al.. 2016 (6) 0.02 [−0.10, 0.15]
Sam et al.. 2016 (7) 0.62 [0.53, 0.69]
Sam et al.. 2016 (8) 0.21 [0.09, 0.33]
Sam et al.. 2016 (9) 0.61 [0.52, 0.68]
Sam et al.. 2016 (10) 0.60 [0.51, 0.67]
Sam et al.. 2016 (11) 0.63 [0.55, 0.70]
Gatew et al.. 2017 (1) 0.61 [0.56, 0.66]
Gatew et al.. 2017 (2) 0.62 [0.57, 0.67]
Gatew et al.. 2017 (3) 0.80 [0.77, 0.83]
Gatew et al.. 2017 (4) 0.67 [0.62, 0.71]
Gatew et al.. 2017 (5) 0.46 [0.39, 0.52]
Gatew et al.. 2017 (6) 0.25 [0.17, 0.32]
Zergaw et al.. 2017 (1) 0.84 [0.81, 0.86]
Zergaw et al.. 2017 (2) 0.62 [0.56, 0.67]
Dakhlan et al.. 2020 0.74 [0.57, 0.86]
Karna et al.. 2020 (1) 0.85 [0.83, 0.87]
Karna et al.. 2020 (2) 0.76 [0.73, 0.78]
Karna et al.. 2020 (3) 0.75 [0.72, 0.78]
Karna et al.. 2020 (4) 0.64 [0.60, 0.67]
Mulai et al.. 2020 (1) 0.90 [0.88, 0.91]
Mulai et al.. 2020 (2) 0.59 [0.54, 0.64]
Dakhlan et al.. 2021 (1) 0.86 [0.75, 0.92]
Dakhlan et al.. 2021 (2) 0.72 [0.65, 0.78]
Dakhlan et al.. 2021 (3) 0.73 [0.66, 0.79]
Dakhlan et al.. 2021 (4) 0.82 [0.77, 0.86]
Dakhlan et al.. 2021 (5) 0.48 [0.37, 0.58]
Dakhlan et al.. 2021 (6) 0.67 [0.59, 0.74]
Dakhlan et al.. 2021 (7) 0.83 [0.78, 0.87]
Mathapo and Tyasi. 2021 (1) 0.62 [0.45, 0.75]
Mathapo and Tyasi. 2021 (2) 0.73 [0.59, 0.82]
Ofori et al.. 2021 0.58 [0.52, 0.65]
Ouchene−Khelifi and Ouchene. 2021 0.80 [0.78, 0.82]
Rashijane et al.. 2021 (1) 0.62 [0.45, 0.74]
Rashijane et al.. 2021 (2) 0.86 [0.78, 0.91]
Sheriff et al.. 2021 (1) 0.93 [0.92, 0.94]
Sheriff et al.. 2021 (2) 0.82 [0.80, 0.84]
Tyasi et al.. 2021 (1) 0.77 [0.66, 0.85]
Tyasi et al.. 2021 (2) 0.83 [0.75, 0.89]
Yousuf et al.. 2021 (1) 0.75 [0.72, 0.78]
Yousuf et al.. 2021 (2) 0.75 [0.72, 0.78]
Yousuf et al.. 2021 (3) 0.79 [0.76, 0.81]
Yousuf et al.. 2021 (4) 0.75 [0.72, 0.78]
Getaneh et al.. 2022 (1) 0.52 [0.46, 0.57]
Getaneh et al.. 2022 (2) 0.70 [0.66, 0.74]
Mokoena et al.. 2022 0.93 [0.88, 0.96]
Negesse et al.. 2022 (1) 0.95 [0.94, 0.96]
Negesse et al.. 2022 (2) 0.97 [0.96, 0.97]
Assan et al.. 2023 0.70 [0.45, 0.85]
Dongre et al.. 2023 (1) 0.70 [0.67, 0.73]
Dongre et al.. 2023 (2) 0.75 [0.72, 0.78]
Dongre et al.. 2023 (3) 0.79 [0.77, 0.81]
Dongre et al.. 2023 (4) 0.82 [0.80, 0.84]
Mallam et al.. 2023 0.94 [0.91, 0.96]
Rashijane et al.. 2023 (1) 0.90 [0.87, 0.92]
Rashijane et al.. 2023 (2) 0.72 [0.64, 0.78]
Tyasi and Celik. 2023 (1) 0.64 [0.58, 0.69]
Tyasi and Celik. 2023 (2) 0.58 [0.51, 0.64]
Zewdie et al.. 2023 (1) 0.89 [0.87, 0.91]
Zewdie et al.. 2023 (2) 0.87 [0.85, 0.89]
Lendrawati et al.. 2024 0.72 [0.61, 0.81]
Lestari et al.. 2024 (1) 0.46 [0.35, 0.56]
Lestari et al.. 2024 (2) 0.32 [0.19, 0.44]
Taye et al.. 2024 (1) 0.80 [0.77, 0.83]
Taye et al.. 2024 (2) 0.70 [0.65, 0.74]
Ahmed et al.. 2025 0.84 [0.71, 0.91]
Kegne et al.. 2025 (1) 0.86 [0.84, 0.88]
Kegne et al.. 2025 (2) 0.79 [0.76, 0.82]
Shabangu et al.. 2025 (1) 0.98 [0.97, 0.99]
Shabangu et al.. 2025 (2) 0.72 [0.57, 0.82]
Tyasi. 2025 (1) 0.37 [0.18, 0.53]
Tyasi. 2025 (2) 0.88 [0.83, 0.92]
Tyasi. 2025 (3) 0.84 [0.77, 0.89]
Tyasi. 2025 (4) 0.67 [0.54, 0.76]
Tyasi. 2025 (5) 0.79 [0.70, 0.85]
Tyasi. 2025 (6) 0.53 [0.38, 0.66]
Multilevel random−effects model 0.78 [0.73, 0.83]
−1
−0.5
0
0.5
1
Correlation coefficient (r): Body weight × Body length
