# Supplementary material for: Relationship Between Live Body Weight and Linear Body Measurements in Goats: A Meta-Analysis Using a Multilevel Random-Effects Model
Source: Animals (Basel). 2026 Oct 2;16(19):3107. doi: 10.3390/ani16193107 (PMC13663463; doi:10.3390/ani16193107)
Supplement: Supplementary file 1 [file animals-16-03107-s001.zip › Figure S3.pdf]

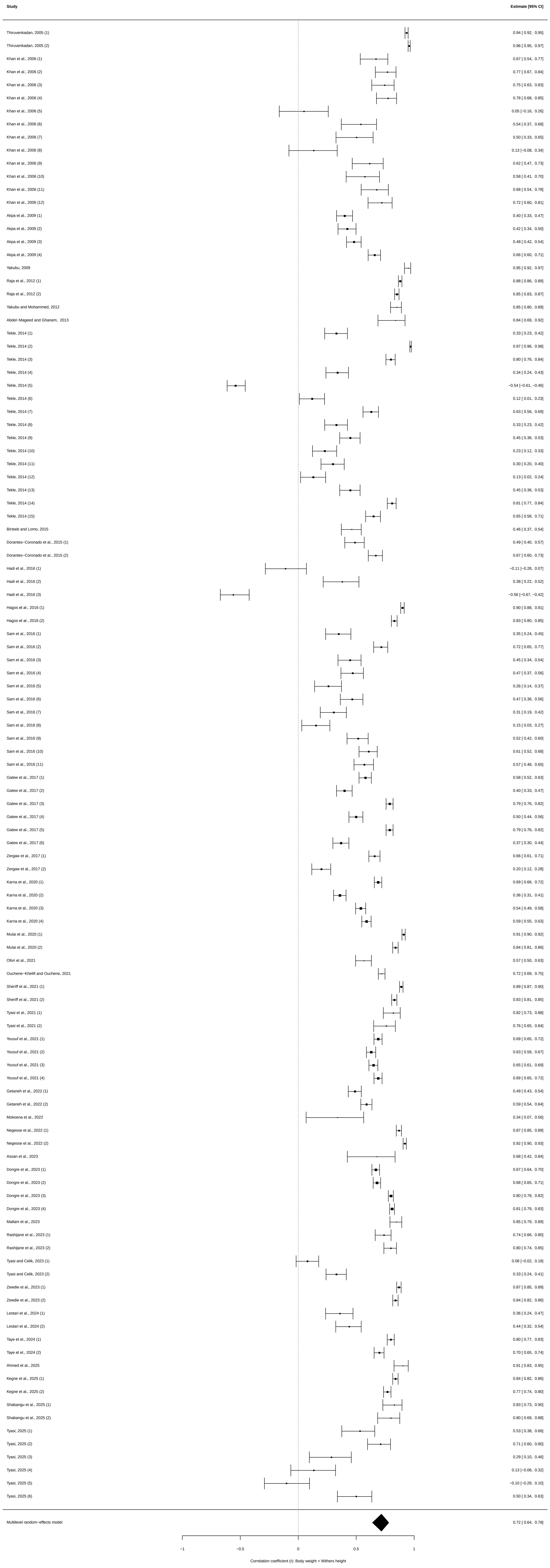

Study
Estimate [95% CI]
Thiruvenkadan, 2005 (1) 0.94 [0.92, 0.95]
Thiruvenkadan, 2005 (2) 0.96 [0.95, 0.97]
Khan et al., 2006 (1) 0.67 [0.54, 0.77]
Khan et al., 2006 (2) 0.77 [0.67, 0.84]
Khan et al., 2006 (3) 0.75 [0.63, 0.83]
Khan et al., 2006 (4) 0.78 [0.68, 0.85]
Khan et al., 2006 (5) 0.05 [−0.16, 0.26]
Khan et al., 2006 (6) 0.54 [0.37, 0.68]
Khan et al., 2006 (7) 0.50 [0.33, 0.65]
Khan et al., 2006 (8) 0.13 [−0.08, 0.34]
Khan et al., 2006 (9) 0.62 [0.47, 0.73]
Khan et al., 2006 (10) 0.58 [0.41, 0.70]
Khan et al., 2006 (11) 0.68 [0.54, 0.78]
Khan et al., 2006 (12) 0.72 [0.60, 0.81]
Akpa et al., 2009 (1) 0.40 [0.33, 0.47]
Akpa et al., 2009 (2) 0.42 [0.34, 0.50]
Akpa et al., 2009 (3) 0.48 [0.42, 0.54]
Akpa et al., 2009 (4) 0.66 [0.60, 0.71]
Yakubu, 2009 0.95 [0.92, 0.97]
Raja et al., 2012 (1) 0.88 [0.86, 0.89]
Raja et al., 2012 (2) 0.85 [0.83, 0.87]
Yakubu and Mohammed, 2012 0.85 [0.80, 0.89]
Abdel−Mageed and Ghanem, 2013 0.84 [0.69, 0.92]
Tekle, 2014 (1) 0.33 [0.23, 0.42]
Tekle, 2014 (2) 0.97 [0.96, 0.98]
Tekle, 2014 (3) 0.80 [0.76, 0.84]
Tekle, 2014 (4) 0.34 [0.24, 0.43]
Tekle, 2014 (5) −0.54 [−0.61, −0.46]
Tekle, 2014 (6) 0.12 [0.01, 0.23]
Tekle, 2014 (7) 0.63 [0.56, 0.69]
Tekle, 2014 (8) 0.33 [0.23, 0.42]
Tekle, 2014 (9) 0.45 [0.36, 0.53]
Tekle, 2014 (10) 0.23 [0.12, 0.33]
Tekle, 2014 (11) 0.30 [0.20, 0.40]
Tekle, 2014 (12) 0.13 [0.02, 0.24]
Tekle, 2014 (13) 0.45 [0.36, 0.53]
Tekle, 2014 (14) 0.81 [0.77, 0.84]
Tekle, 2014 (15) 0.65 [0.58, 0.71]
Birteeb and Lomo, 2015 0.46 [0.37, 0.54]
Dorantes−Coronado et al., 2015 (1) 0.49 [0.40, 0.57]
Dorantes−Coronado et al., 2015 (2) 0.67 [0.60, 0.73]
Hadi et al., 2016 (1) −0.11 [−0.28, 0.07]
Hadi et al., 2016 (2) 0.38 [0.22, 0.52]
Hadi et al., 2016 (3) −0.56 [−0.67, −0.42]
Hagos et al., 2016 (1) 0.90 [0.88, 0.91]
Hagos et al., 2016 (2) 0.83 [0.80, 0.85]
Sam et al., 2016 (1) 0.35 [0.24, 0.45]
Sam et al., 2016 (2) 0.72 [0.65, 0.77]
Sam et al., 2016 (3) 0.45 [0.34, 0.54]
Sam et al., 2016 (4) 0.47 [0.37, 0.56]
Sam et al., 2016 (5) 0.26 [0.14, 0.37]
Sam et al., 2016 (6) 0.47 [0.36, 0.56]
Sam et al., 2016 (7) 0.31 [0.19, 0.42]
Sam et al., 2016 (8) 0.15 [0.03, 0.27]
Sam et al., 2016 (9) 0.52 [0.42, 0.60]
Sam et al., 2016 (10) 0.61 [0.52, 0.68]
Sam et al., 2016 (11) 0.57 [0.48, 0.65]
Gatew et al., 2017 (1) 0.58 [0.52, 0.63]
Gatew et al., 2017 (2) 0.40 [0.33, 0.47]
Gatew et al., 2017 (3) 0.79 [0.76, 0.82]
Gatew et al., 2017 (4) 0.50 [0.44, 0.56]
Gatew et al., 2017 (5) 0.79 [0.76, 0.82]
Gatew et al., 2017 (6) 0.37 [0.30, 0.44]
Zergaw et al., 2017 (1) 0.66 [0.61, 0.71]
Zergaw et al., 2017 (2) 0.20 [0.12, 0.28]
Karna et al., 2020 (1) 0.69 [0.66, 0.72]
Karna et al., 2020 (2) 0.36 [0.31, 0.41]
Karna et al., 2020 (3) 0.54 [0.49, 0.58]
Karna et al., 2020 (4) 0.59 [0.55, 0.63]
Mulai et al., 2020 (1) 0.91 [0.90, 0.92]
Mulai et al., 2020 (2) 0.84 [0.81, 0.86]
Ofori et al., 2021 0.57 [0.50, 0.63]
Ouchene−Khelifi and Ouchene, 2021 0.72 [0.69, 0.75]
Sheriff et al., 2021 (1) 0.89 [0.87, 0.90]
Sheriff et al., 2021 (2) 0.83 [0.81, 0.85]
Tyasi et al., 2021 (1) 0.82 [0.73, 0.88]
Tyasi et al., 2021 (2) 0.76 [0.65, 0.84]
Yousuf et al., 2021 (1) 0.69 [0.65, 0.72]
Yousuf et al., 2021 (2) 0.63 [0.59, 0.67]
Yousuf et al., 2021 (3) 0.65 [0.61, 0.69]
Yousuf et al., 2021 (4) 0.69 [0.65, 0.72]
Getaneh et al., 2022 (1) 0.49 [0.43, 0.54]
Getaneh et al., 2022 (2) 0.59 [0.54, 0.64]
Mokoena et al., 2022 0.34 [0.07, 0.56]
Negesse et al., 2022 (1) 0.87 [0.85, 0.89]
Negesse et al., 2022 (2) 0.92 [0.90, 0.93]
Assan et al., 2023 0.68 [0.42, 0.84]
Dongre et al., 2023 (1) 0.67 [0.64, 0.70]
Dongre et al., 2023 (2) 0.68 [0.65, 0.71]
Dongre et al., 2023 (3) 0.80 [0.78, 0.82]
Dongre et al., 2023 (4) 0.81 [0.79, 0.83]
Mallam et al., 2023 0.85 [0.79, 0.89]
Rashijane et al., 2023 (1) 0.74 [0.66, 0.80]
Rashijane et al., 2023 (2) 0.80 [0.74, 0.85]
Tyasi and Celik, 2023 (1) 0.08 [−0.02, 0.18]
Tyasi and Celik, 2023 (2) 0.33 [0.24, 0.41]
Zewdie et al., 2023 (1) 0.87 [0.85, 0.89]
Zewdie et al., 2023 (2) 0.84 [0.82, 0.86]
Lestari et al., 2024 (1) 0.36 [0.24, 0.47]
Lestari et al., 2024 (2) 0.44 [0.32, 0.54]
Taye et al., 2024 (1) 0.80 [0.77, 0.83]
Taye et al., 2024 (2) 0.70 [0.65, 0.74]
Ahmed et al., 2025 0.91 [0.83, 0.95]
Kegne et al., 2025 (1) 0.84 [0.82, 0.86]
Kegne et al., 2025 (2) 0.77 [0.74, 0.80]
Shabangu et al., 2025 (1) 0.83 [0.73, 0.90]
Shabangu et al., 2025 (2) 0.80 [0.69, 0.88]
Tyasi, 2025 (1) 0.53 [0.38, 0.66]
Tyasi, 2025 (2) 0.71 [0.60, 0.80]
Tyasi, 2025 (3) 0.29 [0.10, 0.46]
Tyasi, 2025 (4) 0.13 [−0.06, 0.32]
Tyasi, 2025 (5) −0.10 [−0.29, 0.10]
Tyasi, 2025 (6) 0.50 [0.34, 0.63]
Multilevel random−effects model 0.72 [0.64, 0.78]
−1
−0.5
0
0.5
1
Correlation coefficient (r): Body weight × Withers height
