# Supplementary material for: Relationship Between Live Body Weight and Linear Body Measurements in Goats: A Meta-Analysis Using a Multilevel Random-Effects Model
Source: Animals (Basel). 2026 Oct 2;16(19):3107. doi: 10.3390/ani16193107 (PMC13663463; doi:10.3390/ani16193107)
Supplement: Supplementary file 1 [file animals-16-03107-s001.zip › Table S1.pdf]

Table S1. Search strings and syntax used for database querying

Database	Platform / Interface	Search Restrictions	String	Hits Retrieved
**PubMed**	NLM / NCBI	[Title/Abstract]	(("goat"[Title/Abstract] OR "goats"[Title/Abstract] OR "caprine"[Title/Abstract]) AND ("body weight"[Title/Abstract] OR "live weight"[Title/Abstract] OR "body weight prediction"[Title/Abstract]) AND ("heart girth"[Title/Abstract] OR "chest girth"[Title/Abstract] OR "thoracic girth"[Title/Abstract] OR "linear body measurements"[Title/Abstract] OR "body length"[Title/Abstract] OR "withers height"[Title/Abstract]) AND ("correlation"[Title/Abstract] OR "regression"[Title/Abstract] OR "prediction"[Title/Abstract]))	32
**Web of Science**	Clarivate (Core Collection)	TS (Topic)	TS=(("goat" OR "goats" OR "caprine") AND ("body weight" OR "live weight" OR "body weight prediction") AND ("heart girth" OR "chest girth" OR "thoracic girth" OR "linear body measurements" OR "body length" OR "withers height") AND ("correlation" OR "regression" OR "prediction"))	25
**Scopus**	Elsevier	TITLE-ABS-KEY	TITLE-ABS-KEY(("goat" OR "goats" OR "caprine") AND ("body weight" OR "live weight" OR "body weight prediction") AND ("heart girth" OR "chest girth" OR "thoracic girth" OR "linear body measurements" OR "body length" OR "withers height") AND ("correlation" OR "regression" OR "prediction"))	140
**OpenAlex**	OpenAlex API / Search	Title & Abstract	(title_and_abstract.search: ("goat" OR "goats" OR "caprine")) AND (title_and_abstract.search: ("body weight" OR "live weight" OR "body weight prediction")) AND (title_and_abstract.search: ("heart girth" OR "chest girth" OR "thoracic girth" OR "linear body measurements" OR "body length" OR "withers height")) AND (title_and_abstract.search: ("correlation" OR "regression" OR "prediction"))	89
**Google Scholar**	Google	Title / All Fields.	intitle: goat "body weight" "heart girth" OR "linear body measurements" OR "withers height" OR "body length" *(Run alongside key variants:* "body weight prediction" goat, *`"live weight" "heart girth" goat)*	20
