# Supplementary material for: Relationship Between Live Body Weight and Linear Body Measurements in Goats: A Meta-Analysis Using a Multilevel Random-Effects Model
Source: Animals (Basel). 2026 Oct 2;16(19):3107. doi: 10.3390/ani16193107 (PMC13663463; doi:10.3390/ani16193107)
Supplement: Supplementary file 1 [file animals-16-03107-s001.zip › Table S2.pdf]

Table S2. JBI critical appraisal of the methodological quality of the studies

**Author**	**Q1**	**Q2**	**Q3**	**Q4**	**Q5**	**Q6**	**Q7**	**Q8**	**Yes/8**	**Risk**
Abdel-Mageed and Ghanem, 2013 [15]	No	Yes	Yes	Yes	Yes	Yes	Yes	Yes	7	Low
Ahmed et al., 2025 [47]	No	Yes	Yes	Yes	No	No	Yes	Yes	6	Low
Akpa et al., 2009 [10]	Yes	Yes	Yes	Yes	Yes	Yes	Yes	Yes	8	Low
Assan et al., 2023 [39]	Yes	Yes	Yes	Yes	Yes	Yes	Yes	Yes	8	Low
Birteeb and Lomo, 2015 [17]	Yes	Yes	Yes	Yes	No	No	Yes	Yes	6	Low
Dakhlan et al., 2020 [24]	Yes	Yes	Yes	Yes	Yes	Yes	Yes	Yes	8	Low
Dakhlan et al., 2021a [27]	Yes	Yes	Yes	Yes	Yes	Yes	Yes	Yes	8	Low
Dakhlan et al., 2021b [28]	Yes	Yes	Yes	Yes	Yes	Yes	Yes	Yes	8	Low
Dongre et al., 2023 [40]	Yes	Yes	Yes	Yes	Yes	Yes	Yes	Yes	8	Low
Dorantes-Coronado et al., 2015 [18]	Yes	Yes	Yes	Yes	Yes	Yes	Yes	Yes	8	Low
Gatew et al., 2017 [22]	Yes	Yes	Yes	Yes	Yes	Yes	Yes	Yes	8	Low
Getaneh et al., 2022 [36]	Yes	Yes	Yes	Yes	Yes	Yes	Yes	Yes	8	Low
Hadi et al., 2016 [19]	Yes	Yes	Yes	Yes	Yes	Yes	Yes	Yes	8	Low
Hagos et al., 2016 [20]	Yes	Yes	Yes	Yes	Yes	Yes	Yes	Yes	8	Low
Karna et al., 2020 [25]	Yes	Yes	Yes	Yes	Yes	Yes	Yes	Yes	8	Low
Kegne et al., 2025 [48]	Yes	Yes	Yes	Yes	Yes	Yes	Yes	Yes	8	Low
Khan et al., 2006 [9]	Yes	Yes	Yes	Yes	Yes	Yes	Yes	No	7	Low
Lendrawati et al., 2024 [44]	Yes	Yes	Yes	Yes	No	No	Yes	Yes	6	Low
Lestari et al., 2024 [45]	Yes	Yes	Yes	Yes	No	No	Yes	Yes	6	Low
Mallam et al., 2023 [41]	Yes	Yes	Yes	Yes	Yes	Yes	Yes	Yes	8	Low
Mathapo and Tyasi, 2021 [29]	Yes	Yes	Yes	Yes	Yes	Yes	Yes	Yes	8	Low
Mokoena et al., 2022 [2]	No	Yes	Yes	Yes	Yes	Yes	Yes	Yes	7	Low
Mulai et al., 2020 [26]	Yes	Yes	Yes	Yes	Yes	Yes	Yes	Yes	8	Low
Muluneh and Tadesse, 2022 [37]	Yes	Yes	Yes	Yes	Yes	Yes	Yes	Yes	8	Low
Negesse et al., 2022 [38]	Yes	Yes	Yes	Yes	Yes	Yes	Yes	Yes	8	Low
Ofori et al., 2021 [30]	Yes	Yes	Yes	Yes	Yes	Yes	Yes	Yes	8	Low
Ouchene-Khelifi and Ouchene, 2021 [31]	No	Yes	Yes	Yes	Yes	Yes	Yes	Yes	7	Low
Raja et al., 2012 [13]	No	Yes	Yes	Yes	Yes	Yes	Yes	Yes	7	Low
Rashijane et al., 2021 [32]	Yes	Yes	Yes	Yes	Yes	No	Yes	Yes	7	Low

Rashijane et al., 2023 [3]	Yes	Yes	Yes	Yes	No	No	Yes	Yes	6	Low
Sam et al., 2016 [21]	Yes	Yes	Yes	Yes	Yes	Yes	Yes	Yes	8	Low
Shabangu et al., 2025 [49]	Yes	Yes	Yes	Yes	Yes	Yes	Yes	Yes	8	Low
Sheriff et al., 2021 [33]	Yes	Yes	Yes	Yes	Yes	Yes	Yes	Yes	8	Low
Taye et al., 2024 [46]	Yes	Yes	Yes	Yes	Yes	Yes	Yes	Yes	8	Low
Tekle, 2014 [16]	Yes	Yes	Yes	Yes	Yes	Yes	Yes	Yes	8	Low
Thiruvenkadan, 2005 [8]	Yes	Yes	Yes	Yes	Yes	Yes	Yes	Yes	8	Low
Tyasi and Celik, 2023 [42]	Yes	Yes	Yes	Yes	Yes	Yes	No	Yes	7	Low
Tyasi et al., 2021 [34]	Yes	Yes	Yes	Yes	Yes	Yes	No	Yes	7	Low
Tyasi, 2025 [50]	Yes	Yes	Yes	Yes	Yes	Yes	Yes	Yes	8	Low
Yakubu and Mohammed, 2012 [14]	Yes	Yes	Yes	Yes	Yes	Yes	Yes	Yes	8	Low
Yakubu et al., 2011 [12]	Yes	Yes	Yes	Yes	Yes	Yes	Yes	Yes	8	Low
Yakubu, 2009 [11]	Yes	Yes	Yes	Yes	Yes	Yes	Yes	Yes	8	Low
Yousuf et al., 2021 [35]	Yes	Yes	Yes	Yes	Yes	Yes	Yes	Yes	8	Low
Zergaw et al., 2017 [23]	Yes	Yes	Yes	Yes	Yes	Yes	Yes	Yes	8	Low
Zewdie et al., 2023 [43]	Yes	Yes	Yes	Yes	Yes	Yes	Yes	Yes	8	Low

Q1–Q8 correspond respectively to: (Q1) clearly defined inclusion criteria; (Q2) detailed description of subjects and setting; (Q3) valid and reliable measurement; (Q4) objective/standard measurement criteria; (Q5) identification of confounders; (Q6) strategies to address confounding; (Q7) valid and reliable outcome measurement; and (Q8) appropriate statistical analysis.
