# Supplementary material for: Relationship Between Live Body Weight and Linear Body Measurements in Goats: A Meta-Analysis Using a Multilevel Random-Effects Model
Source: Animals (Basel). 2026 Oct 2;16(19):3107. doi: 10.3390/ani16193107 (PMC13663463; doi:10.3390/ani16193107)
Supplement: Supplementary file 1 [file animals-16-03107-s001.zip › Table S3.pdf]

Table S3. Leave-one-publication-out sensitivity analysis for the correlation between body weight and heart girth

**Publication excluded**	**Pooled *r***	**95% CI**
Thiruvenkadan (2005) [8]	0.8289	0.7833–0.8656
Khan et al. (2006) [9]	0.8385	0.7945–0.8737
Akpa et al. (2009) [10]	0.8408	0.7989–0.8745
Yakubu (2009) [11]	0.8317	0.7855–0.8686
Raja et al. (2012) [13]	0.8321	0.7858–0.8691
Yakubu and Mohammed (2012) [14]	0.8320	0.7857–0.8690
Abdel-Mageed and Ghanem (2013) [15]	0.8323	0.7862–0.8692
Tekle (2014) [16]	0.8373	0.7924–0.8731
Birteeb and Lomo (2015) [17]	0.8372	0.7924–0.8731
Dorantes-Coronado et al. (2015) [18]	0.8356	0.7899–0.8720
Hadi et al. (2016) [19]	0.8384	0.7945–0.8737
Hagos (2016) [20]	0.8307	0.7846–0.8677
Sam et al. (2016) [21]	0.8379	0.7934–0.8734
Gatew et al. (2017) [22]	0.8347	0.7887–0.8714
Dakhlan et al. (2020) [24]	0.8343	0.7883–0.8709
Karna et al. (2020) [25]	0.8353	0.7895–0.8718
Mulai et al. (2020) [26]	0.8327	0.7864–0.8697
Dakhlan et al. (2021a) [27]	0.8297	0.7839–0.8665
Dakhlan et al. (2021b) [28]	0.8341	0.7880–0.8709
Mathapo and Tyasi (2021) [29]	0.8364	0.7912–0.8726
Ofori et al. (2021) [30]	0.8389	0.7953–0.8739
Ouchene-Khelifi and Ouchene (2021) [31]	0.8313	0.7851–0.8683
Rashijane et al. (2021) [32]	0.8336	0.7875–0.8705
Sheriff et al. (2021) [33]	0.8305	0.7844–0.8675
Tyasi et al. (2021) [34]	0.8361	0.7908–0.8724
Yousuf et al. (2021) [35]	0.8346	0.7886–0.8713
Getaneh et al. (2022) [36]	0.8365	0.7913–0.8726
Mokoena et al. (2022) [2]	0.8332	0.7870–0.8701
Muluneh and Tadesse (2022) [37]	0.8352	0.7894–0.8717
Negesse et al. (2022) [38]	0.8280	0.7828–0.8646
Assan et al. (2023) [39]	0.8352	0.7895–0.8716
Dongre et al. (2023) [40]	0.8363	0.7910–0.8725
Mallam et al. (2023) [41]	0.8310	0.7849–0.8680
Rashijane et al. (2023) [3]	0.8317	0.7854–0.8687
Tyasi and Celik (2023) [42]	0.8418	0.8012–0.8747
Zewdie et al. (2023) [43]	0.8314	0.7852–0.8684
Lendrawati et al. (2024) [44]	0.8348	0.7889–0.8714
Lestari et al. (2024) [45]	0.8381	0.7938–0.8735
Taye et el. (2024) [46]	0.8337	0.7876–0.8706
Ahmed et al. (2025) [47]	0.8317	0.7855–0.8686
Kegne et al. (2025) [48]	0.8334	0.7872–0.8703
Shabangu et al. (2025) [49]	0.8305	0.7844–0.8674
Tyasi (2025) [50]	0.8366	0.7914–0.8727

r: correlation
