# Supplementary material for: Relationship Between Live Body Weight and Linear Body Measurements in Goats: A Meta-Analysis Using a Multilevel Random-Effects Model
Source: Animals (Basel). 2026 Oct 2;16(19):3107. doi: 10.3390/ani16193107 (PMC13663463; doi:10.3390/ani16193107)
Supplement: Supplementary file 1 [file animals-16-03107-s001.zip › Table S4.pdf]

Table S4. Leave-one-publication-out sensitivity analysis for the correlation between body weight and body length

**Publication excluded**	**Pooled *r***	**95% CI**
Negesse et al., 2022 [38]	0.7772	0.7268–0.8192
Thiruvenkadan, 2005 [8]	0.7787	0.7274–0.8213
Hadi et al., 2016 [19]	0.7931	0.7449–0.8332
Akpa et al., 2009 [10]	0.7928	0.7441–0.8331
Mallam et al., 2023 [41]	0.7798	0.7281–0.8227
Lestari et al., 2024 [45]	0.7919	0.7425–0.8328
Sam et al., 2016 [21]	0.7916	0.7418–0.8326
Yakubu, 2009 [11]	0.7809	0.7289–0.8240
Mokoena et al., 2022 [2]	0.7810	0.7289–0.8241
Shabangu et al., 2025 [49]	0.7811	0.7289–0.8243
Yakubu and Mohammed, 2012 [14]	0.7812	0.7290–0.8244
Khan et al., 2006 [9]	0.7905	0.7400–0.8322
Ofori et al., 2021 [30]	0.7897	0.7386–0.8317
Gatew et al., 2017 [22]	0.7896	0.7385–0.8317
Hagos, 2016 [20]	0.7826	0.7301–0.8260
Tyasi and Celik, 2023 [42]	0.7893	0.7381–0.8315
Sheriff et al., 2021 [33]	0.7828	0.7302–0.8261
Getaneh et al., 2022 [36]	0.7892	0.7379–0.8315
Abdel-Mageed and Ghanem, 2013 [15]	0.7829	0.7306–0.8261
Zewdie et al., 2023 [43]	0.7831	0.7305–0.8264
Tekle, 2014 [16]	0.7885	0.7369–0.8310
Raja et al., 2012 [13]	0.7838	0.7312–0.8272
Dorantes-Coronado et al., 2015 [18]	0.7883	0.7365–0.8309
Mathapo and Tyasi, 2021 [29]	0.7882	0.7365–0.8308
Dakhlan et al., 2021a [27]	0.7842	0.7318–0.8275
Birteeb and Lomo, 2015 [17]	0.7878	0.7358–0.8305
Assan et al., 2023 [39]	0.7876	0.7358–0.8302
Tyasi, 2025 [50]	0.7875	0.7355–0.8303
Dakhlan et al., 2021b [28]	0.7874	0.7353–0.8303
Lendrawati et al., 2024 [44]	0.7874	0.7354–0.8302
Rashijane et al., 2023 [3]	0.7849	0.7323–0.8282
Ahmed et al., 2025 [47]	0.7849	0.7325–0.8281
Kegne et al., 2025 [48]	0.7849	0.7323–0.8282
Zergaw et al., 2017 [23]	0.7869	0.7347–0.8299
Dakhlan et al., 2020 [24]	0.7869	0.7348–0.8298
Taye et al., 2024 [46]	0.7868	0.7346–0.8298
Karna et al., 2020 [25]	0.7867	0.7344–0.8297
Yousuf et al., 2021 [35]	0.7867	0.7344–0.8297
Rashijane et al., 2021 [32]	0.7866	0.7343–0.8296
Dongre et al., 2023 [40]	0.7865	0.7341–0.8296
Ouchene-Khelifi and Ouchene, 2021 [31]	0.7856	0.7331–0.8289
Tyasi et al., 2021 [34]	0.7857	0.7332–0.8289
Mulai et al., 2020 [26]	0.7860	0.7335–0.8291

r: correlation coefficient
