# Supplementary material for: Relationship Between Live Body Weight and Linear Body Measurements in Goats: A Meta-Analysis Using a Multilevel Random-Effects Model
Source: Animals (Basel). 2026 Oct 2;16(19):3107. doi: 10.3390/ani16193107 (PMC13663463; doi:10.3390/ani16193107)
Supplement: Supplementary file 1 [file animals-16-03107-s001.zip › Table S5.pdf]

Table S5. Leave-one-publication-out sensitivity analysis for the correlation between body weight and withers height

**Publication excluded**	**Pooled *r***	**95% CI**
Thiruvenkadan (2005) [8]	0.7049	0.6285–0.7678
Khan et al. (2006) [9]	0.7208	0.6423–0.7843
Akpa et al. (2009) [10]	0.7228	0.6454–0.7856
Yakubu (2009) [11]	0.7058	0.6290–0.7690
Raja et al. (2012) [13]	0.7121	0.6326–0.7767
Yakubu and Mohammed (2012) [11]	0.7131	0.6335–0.7778
Abdel-Mageed and Ghanem (2013) [15]	0.7142	0.6348–0.7786
Tekle (2014) [16]	0.7229	0.6455–0.7856
Birteeb & Lomo (2015) [17]	0.7233	0.6461–0.7858
Dorantes-Coronado et al. (2015) [18]	0.7210	0.6427–0.7844
Hadi et al. (2016) [19]	0.7312	0.6614–0.7884
Hagos (2016) [20]	0.7119	0.6324–0.7765
Sam et al. (2016) [21]	0.7234	0.6464–0.7859
Gatew et al. (2017) [22]	0.7207	0.6423–0.7843
Zergaw et al. (2017) [23]	0.7234	0.6463–0.7859
Karna et al. (2020) [25]	0.7217	0.6436–0.7849
Mulai et al. (2020) [26]	0.7113	0.6319–0.7759
Ofori et al. (2021) [30]	0.7214	0.6432–0.7847
Ouchene-Khelifi and Ouchene (2021) [31]	0.7178	0.6384–0.7821
Sheriff et al. (2021) [33]	0.7123	0.6327–0.7769
Tyasi et al. (2021) [34]	0.7156	0.6359–0.7801
Yousuf et al. (2021) [35]	0.7193	0.6402–0.7832
Getaneh et al. (2022) [36]	0.7219	0.6440–0.7850
Mokoena et al. (2022) [2]	0.7246	0.6485–0.7863
Negesse et al. (2022) [38]	0.7100	0.6311–0.7745
Assan et al. (2023) [39]	0.7187	0.6399–0.7826
Dongre et al. (2023) [40]	0.7170	0.6374–0.7815
Mallam et al. (2023) [41]	0.7132	0.6336–0.7778
Rashijane et al. (2023) [3]	0.7162	0.6366–0.7808
Tyasi and Celik (2023) [42]	0.7271	0.6529–0.7875
Zewdie et al. (2023) [43]	0.7127	0.6331–0.7773
Lestari et al. (2024) [45]	0.7243	0.6478–0.7863
Taye et al. (2024) [46]	0.7168	0.6372–0.7813
Ahmed et al. (2025) [47]	0.7105	0.6317–0.7748
Kegne et al. (2025) [48]	0.7149	0.6351–0.7795
Shabangu et al. (2025) [49]	0.7147	0.6350–0.7793
Tyasi (2025) [50]	0.7247	0.6485–0.7865

r: Correlation coefficient
